# Reliable prediction of protein–protein binding affinity changes upon mutations with Pythia-PPI

**DOI:** 10.1093/nsr/nwaf231

**Published:** 2025-06-10

**Authors:** Fangting Tao, Jinyuan Sun, Pengyue Gao, George Fu Gao, Bian Wu

**Affiliations:** Division of Life Sciences and Medicine, University of Science and Technology of China, Hefei 230026, China; Laboratory of Pathogen Microbiology and Immunology, Institute of Microbiology, Chinese Academy of Sciences, Beijing 100101, China; AIM center, College of Life Sciences and Technology, Beijing University of Chemical Technology, Institute of Microbiology, Chinese Academy of Sciences, Beijing 100029, China; AIM center, College of Life Sciences and Technology, Beijing University of Chemical Technology, Institute of Microbiology, Chinese Academy of Sciences, Beijing 100029, China; College of Life Sciences, University of Chinese Academy of Sciences, Beijing 100049, China; State Key Laboratory of Microbial Diversity and Innovative Utilization, Institute of Microbiology, Chinese Academy of Sciences, Beijing 100101, China; Division of Life Sciences and Medicine, University of Science and Technology of China, Hefei 230026, China; Laboratory of Pathogen Microbiology and Immunology, Institute of Microbiology, Chinese Academy of Sciences, Beijing 100101, China; Division of Life Sciences and Medicine, University of Science and Technology of China, Hefei 230026, China; Laboratory of Pathogen Microbiology and Immunology, Institute of Microbiology, Chinese Academy of Sciences, Beijing 100101, China; AIM center, College of Life Sciences and Technology, Beijing University of Chemical Technology, Institute of Microbiology, Chinese Academy of Sciences, Beijing 100029, China; State Key Laboratory of Green Biomanufacturing, Beijing University of Chemical Technology, Beijing 100029, China

**Keywords:** AI for microbiology, protein–protein interaction, protein engineering, machine learning

## Abstract

Protein–protein interactions (PPIs) are essential for numerous biological functions and predicting binding affinity changes caused by mutations is crucial for understanding the impact of genetic variation and advancing protein engineering. Although machine-learning-based methods show promise in improving prediction accuracy, limited experimental data remain a significant bottleneck. In this study, we employed multitask learning and self-distillation to overcome the data limitation and improve the accuracy of protein–protein binding affinity prediction. By incorporating a mutation stability prediction task, our model achieved state-of-the-art accuracy on the SKEMPI dataset and was subsequently used to predict binding affinity changes for millions of mutations, generating an expanded dataset for self-distillation. Compared with prevalent methods, Pythia-PPI increased the Pearson's correlation between predictions and experimental data from 0.6447 to 0.7850 on the SKEMPI dataset and from 0.3654 to 0.6050 on the viral-receptor dataset. Experimental validation further confirmed its ability to identify high-affinity mutations on the CB6 antibody in complex with the severe acute respiratory syndrome coronavirus 2 prototype receptor binding domain, with the best single-point mutant among the top 10 predictions showing a 2-fold increase in binding affinity. These findings demonstrate that Pythia-PPI is a valuable tool for analysing the fitness landscape of PPIs. A web server for Pythia-PPI is available at https://pythiappi.wulab.xyz for easy access.

## INTRODUCTION

Proteins, as essential macromolecules, play fundamental roles in various physiological functions within organisms [1]. Biological processes such as signal transduction, immune response and viral adhesion rely on protein–protein interactions (PPIs) [2–4]. Therefore, understanding how mutations alter the thermodynamic properties of PPIs is crucial for gaining insights into pathogenic genetic variations or optimizing therapeutic proteins [5,6]. The strength of PPIs is typically evaluated through binding affinity, commonly defined by the Gibbs free energy (ΔG) [7]. The effect of mutations can be evaluated by measuring the difference in ΔG between the mutant and wild-type, referred to as ΔΔG, which can evaluate the impact of the mutations on PPI binding affinity or protein stability. Although experimental methods including isothermal titration calorimetry [8] and the yeast two-hybrid system [9] can be used to measure ΔΔG values in PPIs, these methods are generally time-consuming and labor-intensive. Given these limitations, computational methods offer an efficient alternative.

Computational methods can be broadly classified into two categories: physics-based methods and machine-learning-based methods. The former, such as Rosetta [10] and FoldX [11], utilize energy functions to evaluate physical interactions such as hydrogen bonds and van der Waals interactions between atoms. Although these methods can provide interpretable results, they require extensive sampling of conformational space and are limited by the accuracy of energy functions due to approximate assumptions in dealing with anisotropic interactions [12]. In contrast to physics-based methods, machine-learning-based methods are primarily data-driven. Although many machine-learning algorithms, including artificial neural networks, incorporate tools from physics, neural networks capture data representations through layers of abstraction and identify complex patterns within data rather than relying on human-defined rules [13,14]. Notably, machine learning, especially deep learning, has facilitated scientific advancements in protein science, such as accurate structure prediction with AlphaFold2 [15] and advancements in computational protein design [16].

There have also been attempts to address the binding affinity prediction challenge by using deep learning. For instance, graph neural networks, such as DDGPred [17], require Rosetta-modeled mutant structures and specific energy terms. Convolutional neural networks, such as Binding Oracle [18], require the voxelization of local protein structures. Beyond these computational requirements, a fundamental challenge in the prediction of ΔΔG lies in the scarcity of labeled data. This scarcity makes it difficult to fully capture the complex structure–function relationships within protein–protein complexes, resulting in a low success rate for antibody optimization. Overcoming data limitations and developing reliable, efficient deep-learning-based ΔΔG predictors for PPIs are essential for future applications.

In this study, we developed Pythia-PPI and achieved significantly higher prediction accuracy of protein–protein binding affinity changes upon mutations. Firstly, a vanilla Pythia-PPI model was fine-tuned from the pretrained Pythia model [19] by using transfer learning and multitask learning [20,21], enabling the simultaneous prediction of mutation impacts on both protein stability and protein–protein binding affinity. By leveraging the efficiency and precision of this baseline model, we generated mutation predictions for all protein interaction interfaces in the SKEMPI dataset [22], expanding the training set for PPI-related mutations to nearly 400 000. With this augmented dataset and multitask learning, Pythia-PPI achieved a Pearson's correlation of 0.7850 on the SKEMPI dataset. Moreover, the model underwent experimental validation by using the clinical CB6 antibody in a complex with the severe acute respiratory syndrome coronavirus 2 (SARS-CoV-2) prototype (PT) receptor binding domain (RBD) [23], confirming its ability to predict single-point mutations that enhance binding affinity, with the best mutant achieving a 2-fold improvement in binding affinity among the top 10 predictions. Additionally, Pythia-PPI provides exceptional throughput, capable of processing >10 000 mutation predictions per minute, facilitating large-scale point mutation analyses across the human interactome. For broader accessibility, predictions for protein–protein complexes can be freely conducted on our web server at https://pythiappi.wulab.xyz and the source code is openly available at https://github.com/Wublab/pythia_ppi.

## RESULTS

### Pythia-PPI architecture

**Figure 1.**
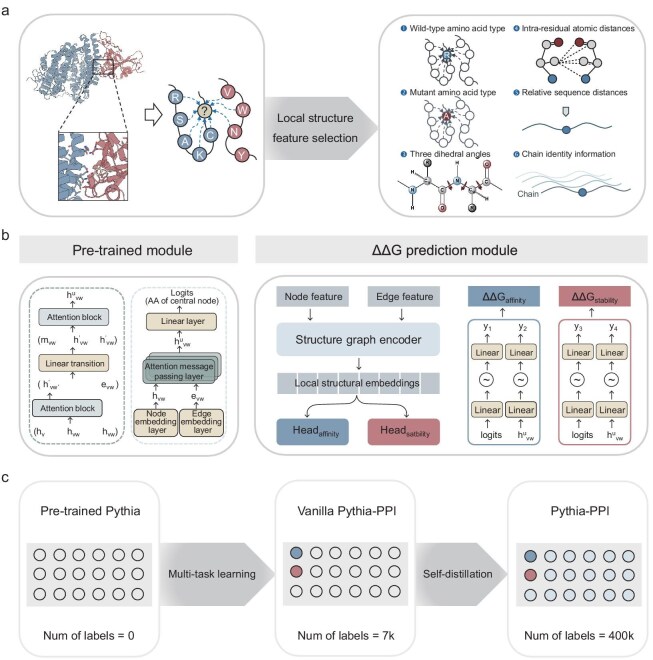
Overview of Pythia-PPI. (a) Pythia-PPI receives a local structure of either a protein–protein complex or a protein as input, depicted as a k-NN graph derived from the Euclidean distances of C-alpha atoms. (b) The architecture of Pythia-PPI, trained on representations extracted from a pretrained model, was aimed at predicting binding affinity changes of PPIs or stability changes of proteins caused by single-point mutations. (c) The process of developing the Pythia-PPI with multitask learning and self-distillation.

Pythia-PPI consists of two modules: a pretrained structure graph encoder module and a ΔΔG prediction module. Employing a k-nearest neighbor (k-NN) graph, Pythia-PPI transforms the local structure of a protein or protein–protein complex into a graph representation (Fig. 1a). Each amino acid acts as a node, establishing connections with its 32 closest amino acids based on the Euclidean distance of the C-alpha atom. The input to the structure graph encoder includes the type of each amino acid encoded via one-hot encoding, while representing backbone dihedral angles (φ, ψ and ω) by using sine and cosine functions as node features. Regarding edge features, we consider distances between the five backbone atoms: C-alpha, C, N, O and C-beta, along with sequence positions and chain information. Node and edge input features are converted into embeddings by the structure graph encoder, identically to the pretrained Pythia. This encoder includes three attention message-passing layers (AMPLs) that generate hidden embeddings and a linear layer that generates probabilities for the 20 amino acids (Fig. 1b).

In Pythia-PPI, hidden embeddings are extracted from each of the AMPLs, along with amino acid probabilities, focusing on the features of both wild-type and mutant residues. Utilizing the message-passing mechanism inherent in Pythia, information about neighboring residues of both wild-type and mutant residues is captured within a structural context. These embeddings are then combined with predicted probabilities from the pretrained module to form the input vector for the ΔΔG prediction module. Pythia-PPI employs a combination of transfer learning and multitask learning, sharing the structure encoder layer across two tasks: predicting changes in PPI binding affinity and protein stability upon mutations. The ΔΔG prediction module comprises two heads, named head_affinity_ and head_stability_, whose selection is determined by the data source of input features. The chosen input vector determines its allocation to either head_affinity_ or head_stability_. Each head consists of two compact multilayer perceptrons, aimed at producing ΔΔG values.

As shown in Fig. 1c, we first evaluated the zero-shot transfer performance of self-supervised models and found that the model learned a potentially better representation with balanced computation costs. Building on this, we improved accuracy in the binding affinity prediction task by multitask supervised fine-tuning. To further enhance the performance of the model, we subsequently applied a self-distillation strategy.

### Supervised fine-tuning largely improved prediction accuracy over zero-shot transferring for binding affinity prediction

**Figure 2.**
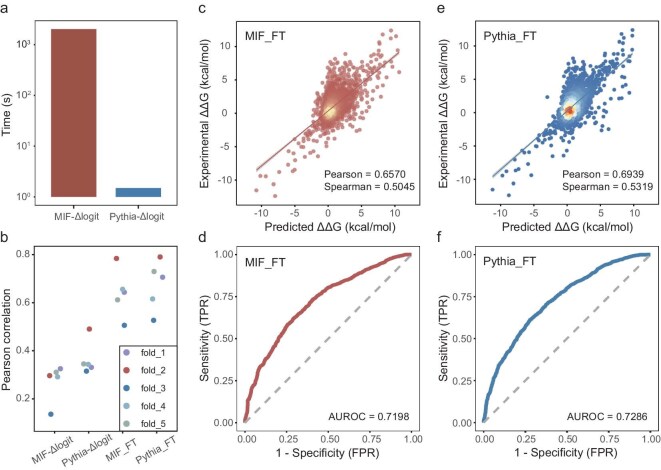
Performance of pretrained and fine-tuned Pythia and MIF. (a) Comparison of computing speed between two methods on all possible single-point mutations based on the PDB structure 6M0J. (b) Pearson's correlation calculated on predictions for models trained on the SKEMPI dataset by 5-fold cross-validation. (c–f) Scatter plots and receiver-operating characteristic (ROC) curves of MIF_FT and Pythia_FT predictions versus experimental values on the SKEMPI dataset; (c) and (d) show MIF_FT results, and (e) and (f) show Pythia_FT results.

Previous studies have demonstrated that protein sequence likelihood models can be effectively generalized to predict the impact of mutations on thermal stability in a zero-shot manner [24]. Our earlier work established a physical relationship between amino acid probability distributions within the structural context of a protein and the thermostability changes associated with protein mutations [19]. Building on this finding, we developed the Pythia model, which achieved state-of-the-art performance in unsupervised thermostability prediction. In this study, we first evaluated Pythia's zero-shot predictive capacity for ∆∆G changes in PPIs, using the SKEMPI dataset as a benchmark. Pythia achieved a Pearson's correlation of 0.3782, outperforming the masked inverse folding (MIF) model [25], which is also a self-supervised structure-aware sequence likelihood model (Table S1). In addition, as depicted in Fig. 2a, Pythia demonstrates significantly faster computational speed compared with MIF, enabling its application in large-scale analyses.

Both Pythia and MIF were fine-tuned on the SKEMPI dataset, resulting in the fine-tuned models Pythia_FT and MIF_FT. After fine-tuning, both models demonstrated improved prediction accuracy for protein–protein binding affinity in 5-fold cross-validation compared with their zero-shot performance (Fig. 2b). When comparing the performance of the two models, Pythia_FT consistently outperformed MIF_FT on the SKEMPI dataset: MIF_FT achieved Pearson's and Spearman's correlations of 0.6570 and 0.5045, respectively, with an area under the receiver-operating characteristic curve (AUROC) of 0.7198 (Fig. 2c and d). In contrast, Pythia_FT achieved enhancements with Pearson's and Spearman's correlations of 0.6939 and 0.5319, respectively, and an AUROC of 0.7286 (Fig. 2e and f). Given Pythia's superior speed, zero-shot transfer performance and fine-tuned prediction accuracy, we selected it as the basis for further multitask predictive model development.

### Multitask learning enhanced the accuracy of structure-wise binding affinity prediction

The prediction of stability changes upon mutations was introduced as an additional training task to enhance the understanding of the relationship between structural and energy changes during the supervised learning. This approach enables the model to learn shared representations of common features between protein structure and thermodynamic parameters from both protein folding stability and protein–protein binding affinity. The FireProtDB contains experimental thermostability data, derived from published datasets and recent literature [26]. This comprehensive database was carefully curated to create the F3436 dataset, which is suitable for machine learning and contains 3436 single-point mutations across 100 proteins [24]. Integrating the F3436 dataset increases the representation of specific mutation types compared with using the SKEMPI dataset (Fig. S1). For training, we combined data from four folds of the SKEMPI dataset with the F3436 dataset, reserving the remaining fold for validation.

**Figure 3.**
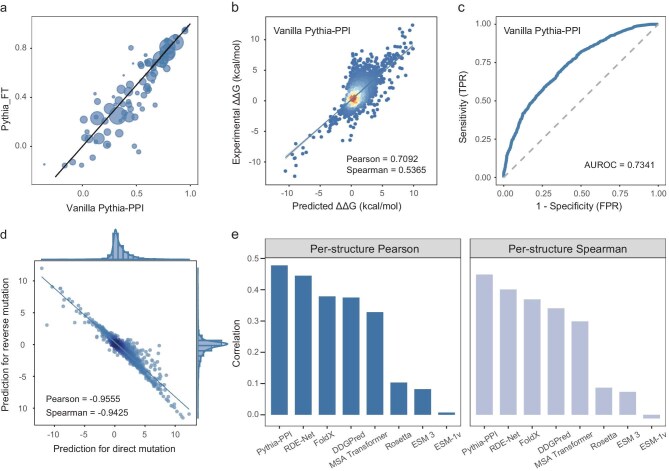
Performance analysis of the vanilla Pythia-PPI on the SKEMPI dataset. (a) Comparison of per-structure Pearson's correlations of Pythia_FT and the vanilla Pythia-PPI. (b) Scatter plot of experimental and predicted ∆∆G values for the vanilla Pythia-PPI. (c) Receiver-operating characteristic (ROC) curve of the vanilla Pythia-PPI. (d) Comparison of predictions for direct and reverse mutations. (e) Bar plot of per-structure Pearson's and Spearman's correlations for tested methods on the SKEMPI dataset.

Through a grid search of stability and affinity loss weights, we identified an optimal affinity-to-stability loss ratio of 0.8:0.2, which maximized the Pearson's correlation (Fig. S2). After incorporating the stability prediction task, the correlations for structure-wise predictions improved noticeably, with the Pearson's correlation increasing from 0.4462 to 0.4778 and the Spearman's correlation increasing from 0.4119 to 0.4486 (Fig. 3a and Table S1). In 5-fold cross-validation on the SKEMPI dataset, the vanilla Pythia-PPI achieved an AUROC of 0.7341 (Fig. 3b), a Pearson's correlation of 0.7092 and a Spearman's correlation of 0.5365 (Fig. 3c). A strong Pearson's correlation of –0.95 between the predicted ∆∆G values of direct and reversed mutations demonstrated that the predictions of our model are almost antisymmetric (Fig. 3d). This indicates that our model has a lower risk of overfitting and improved generalization capability. Compared with other state-of-the-art methods, the vanilla Pythia-PPI improved the per-structure Pearson's correlation from 0.4448 to 0.4778 and the per-structure Spearman's correlation from 0.4010 to 0.4486 (Fig. 3e). This improvement may result from the increased structural diversity in the training data introduced by the stability prediction task, enabling the model to better capture the relationship between protein structure and thermodynamic parameters.

### Data augmentation boosted the prediction accuracy of binding affinity

**Figure 4.**
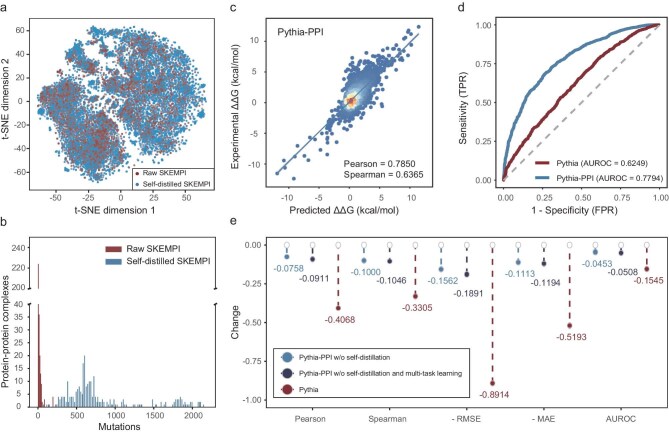
Performance analysis of Pythia-PPI based on the SKEMPI dataset. (a) 2D visualization of the structural distribution of training data in SKEMPI experimental dataset and augmented dataset. (b) Bar plot of mutation numbers versus the corresponding counts of protein-complex structure. (c) Scatter plot of Pythia-PPI predictions versus experimental values. (d) Receiver-operating characteristic (ROC) curve of the final Pythia-PPI and the zero-shot transferred Pythia. (e) Metric reductions for different ablation settings on the SKEMPI dataset.

To further expand the training data and improve the model prediction accuracy and generalization, we adopted a self-distillation strategy. Self-distillation involves training a model by using its own predictions as additional labeled data, which can improve generalization by exposing the model to a broader distribution of examples. We used vanilla Pythia-PPI to predict the ΔΔG for all possible mutations across all amino acids at the interfaces of protein–protein complexes in the SKEMPI dataset. This expansion increased the training dataset for protein–protein binding affinity prediction from ∼4000 to nearly 400 000 samples. When projected into two dimensions by using t-distributed stochastic neighbor embedding (t-SNE) on local structure embeddings from Pythia, the self-distilled samples exhibit both broader and denser spatial coverage compared with the original SKEMPI dataset samples (Fig. 4a). While most complex structures in the original SKEMPI dataset contain only dozens of labeled mutations, the self-distilled dataset contains 200–2000 mutations per complex (Fig. 4b). With this augmented training data, we trained the final Pythia-PPI by using the multitask strategy as previously mentioned.

In benchmark evaluations on the SKEMPI dataset, Pythia-PPI demonstrated robust predictive performance. As shown in Fig. 4c, the model exhibited a Pearson's correlation of 0.7850 between the predicted and experimentally measured ΔΔG values, while achieving a Spearman's correlation of 0.6365 that confirms its ability to accurately capture the relative trends in protein–protein binding affinity. Compared with the baseline model, Pythia-PPI demonstrated substantial improvement in discriminating stable and unstable interactions, while Pythia achieved an AUROC of 0.6249 in zero-shot prediction and our specialized model reached an AUROC of 0.7794 (Fig. 4d). When compared with other state-of-the-art predictors, Pythia-PPI demonstrated superior performance across all key metrics (Table 1). Traditional physics-based approaches, such as Rosetta and FoldX, achieved Pearson's correlations of 0.31, while protein-language models such as ESM-1v [27], ESM 3 [28] and MSA Transformer [29] showed limited predictive power with Pearson's correlations of <0.2. Pythia-PPI outperformed the previous state-of-the-art predictor RDE-Net [30] by improving the overall Pearson's correlation from 0.6447 to 0.7850. The advancement was also evident in per-structure evaluation, where Pythia-PPI achieved a Pearson's correlation of 0.5653 compared with RDE-Net's 0.4448, indicating enhanced generalization across different protein–protein complexes. Beyond correlation metrics, Pythia-PPI also demonstrated superior prediction accuracy with the lowest root mean square error (RMSE) of 1.0796 and mean absolute error (MAE) of 0.7383 among all methods, and achieved the highest discriminative power with an AUROC of 0.7794. These results validate that our integrated approach of combining transfer learning, multitask learning and self-distillation effectively captures the underlying patterns in PPIs. Moreover, Pythia-PPI achieves significantly faster predictions while maintaining comparable accuracy, outperforming both physics-based approaches and several prominent machine-learning-based models (Fig. S4).

**Table 1.** Comparison with various methods for the SKEMPI dataset.

	Per-structure		Overall				
Method	Pearson's	Spearman's	Pearson's	Spearman's	RMSE	MAE	AUROC
Rosetta	0.3284	0.2988	0.3113	0.3468	1.6173	1.1311	0.6562
FoldX	0.3789	0.3693	0.3120	0.4071	1.9080	1.3089	0.6582
ESM-1v	0.0073	–0.0118	0.1921	0.1572	1.9609	1.3683	0.5414
ESM 3	0.0820	0.0734	0.2058	0.1396	4.5945	1.5235	0.5445
MSA Transformer	0.1031	0.0868	0.1173	0.1313	1.9835	1.3816	0.5768
DDGPred	0.3750	0.3407	0.6580	0.4687	1.4998	1.0821	0.6992
RDE-Net	0.4448	0.4010	0.6447	0.5584	1.5799	1.1123	0.7454
Vanilla Pythia-PPI	0.4778	0.4486	0.7092	0.5365	1.2358	0.8496	0.7341
Pythia-PPI	0.5653	0.5271	0.7850	0.6365	1.0796	0.7383	0.7794

To further investigate the contribution of different components in our model-development pipeline, we performed an ablation study and observed that self-distillation substantially improved model performance (Fig. 4e). When compared with the vanilla Pythia-PPI model without self-distillation, the final model achieved an absolute improvement of 0.10 in the Spearman's correlation. More notably, compared with the zero-shot predictions of Pythia, the final Pythia-PPI model demonstrated a remarkable improvement with an absolute increase of 0.41 in the Spearman's correlation, further highlighting the effectiveness of our multitask-learning and data-augmentation strategy.

### Accurate prediction of viral protein fitness and antibody–antigen affinity from complex structures

**Figure 5.**
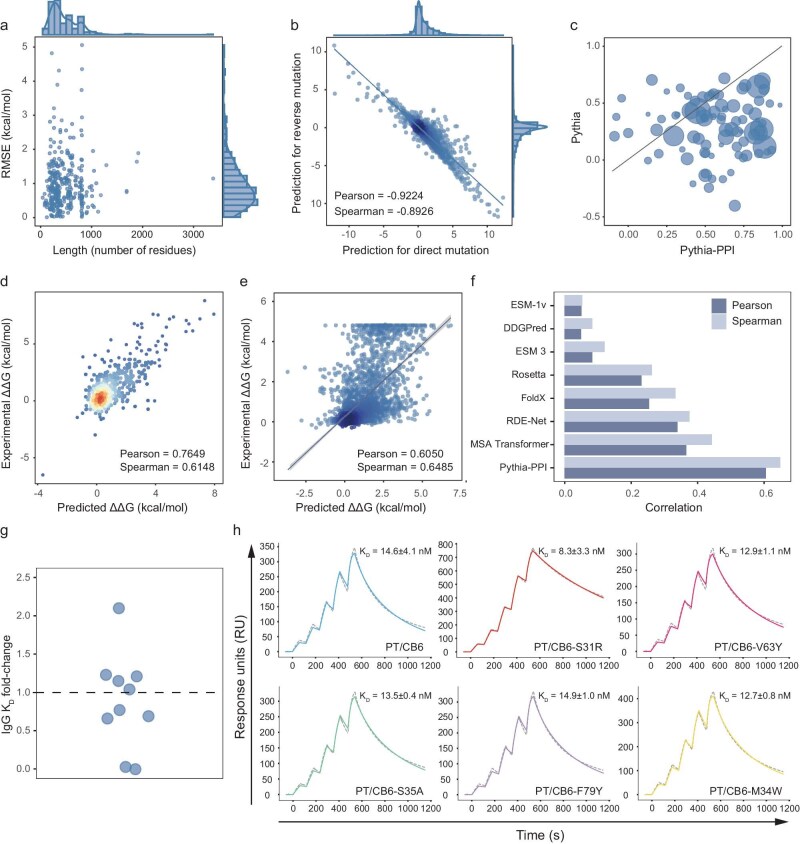
Performance analysis of Pythia-PPI. (a) Scatter plot correlating the per-structure length with the corresponding RMSE on the SKEMPI dataset. (b) Comparison of predictions for direct and reverse mutations based on the SKEMPI dataset. (c) Comparison of per-structure Pearson's values of Pythia and Pythia-PPI based on the SKEMPI dataset. (d) Density scatter plot of Pythia-PPI predictions versus experimental values on the antibody–antigen subset of the SKEMPI dataset. (e) Scatter plot of experimental and predicted ∆∆G on the R3669 dataset. (f) Bar plot of Pearson's and Spearman's correlations for tested methods on the R3669 dataset. (g) Strip plots illustrating CB6 variants, with each point representing a variant plotted based on the fold change in *K*_D_ relative to the wild-type on the *y*-axis and jitter on the *x*-axis. (h) Binding characteristics of the CB6 antibody and its beneficial mutations (CB6-S31R, CB6-V63Y, CB6-S35A, CB6-F79Y and CB6-M34W) with SARS-CoV-2 PT RBD were measured by using surface plasmon resonance (SPR). The *K*_D_ of each is shown as the mean ± SD of three independent experiments.

Examination of RMSE in per-structure predictions revealed that most predictions maintained an RMSE of <2.0 kcal/mol regardless of the size of the protein-complex structures (Fig. 5a). The final model demonstrated strong antisymmetry between direct and reversed mutation predictions, with a Pearson's correlation of –0.9224 and a Spearman's correlation of –0.8926, indicating robust generalization despite extensive self-distillation training (Fig. 5b). Structure-wise comparative analysis showed that Pythia-PPI systematically outperformed Pythia across diverse protein–protein complexes, achieving superior or equivalent Pearson's correlations in 97% of all evaluated structures (Fig. 5c). We also evaluated the performance of Pythia-PPI on antigen–antibody interactions—a key subset of PPIs involved in immune recognition and disease diagnosis. Analysis spanning 588 mutations across 27 antigen–antibody complexes from the SKEMPI dataset demonstrated strong predictive capability, with Pythia-PPI achieving a Pearson's correlation of 0.7649 and Spearman's correlation of 0.6148 (Fig. 5d). In comparison, the vanilla Pythia-PPI model attained correlations of 0.6708 and 0.5263, respectively (Fig. S3).

We also tested Pythia-PPI on a deep mutation scanning dataset for the RBD of SARS-CoV-2 and the human angiotensin-converting enzyme 2 (ACE2) receptor [31]. Based on the reference structure from the protein data bank (PDB) entry 6M0J, we excluded terminal residues to create the R3669 dataset. Pythia-PPI demonstrated superior performance on this independent test set, with Pearson's and Spearman's correlations of 0.6050 and 0.6485, respectively (Fig. 5e). These values markedly surpass the performance of previous state-of-the-art approaches by a significant margin (Table 2). Utilizing evolutionary information, MSA Transformer achieved the highest Pearson's and Spearman's correlations of 0.3654 and 0.4423. In the absence of evolutionary information, the structure-based deep-learning model RDE-Net achieved Pearson's and Spearman's correlations of 0.3395 and 0.3754 (Fig. 5f). These results underscore the potential of Pythia-PPI in antibody binding affinity optimization and viral protein fitness prediction.

**Table 2.** Comparison with other methods on the R3669 dataset.

Method	Pearson's	Spearman's	RMSE	MAE	AUROC
Rosetta	0.2305	0.2624	3.9869	3.1251	0.5970
FoldX	0.2533	0.3336	4.0439	2.2434	0.6293
ESM-1v	0.0505	0.0526	4.8661	4.5936	0.5230
ESM 3	0.0832	0.1201	2.7791	1.1284	0.5769
MSA Transformer	0.3654	0.4423	7.8735	7.3045	0.7528
DDGPred	0.0492	0.0826	1.7700	1.0966	0.4932
RDE-Net	0.3395	0.3754	1.5700	0.9280	0.6516
Pythia-PPI	0.6050	0.6485	1.1836	0.8261	0.7956

In addition to *in silico* evaluations, we conducted experimental validation. According to the predictions of Pythia-PPI, 10 top-ranked single-point mutations in the heavy-chain variable region of the clinical antibody CB6, which was developed for the treatment of SARS-CoV-2 infection [22], were selected. These mutants were synthesized and assessed by using surface plasmon resonance (SPR) measurements [32] (Table S4). Among the 10 mutants tested, the V63Y, S35A, F79Y and M34W mutants demonstrated modest improvements in affinity, while the S31R mutant achieved a significant 2-fold increase (Fig. 5g and h). These results further confirm the reliability of Pythia-PPI in practical applications.

### Development of a user-friendly web server

**Figure 6.**
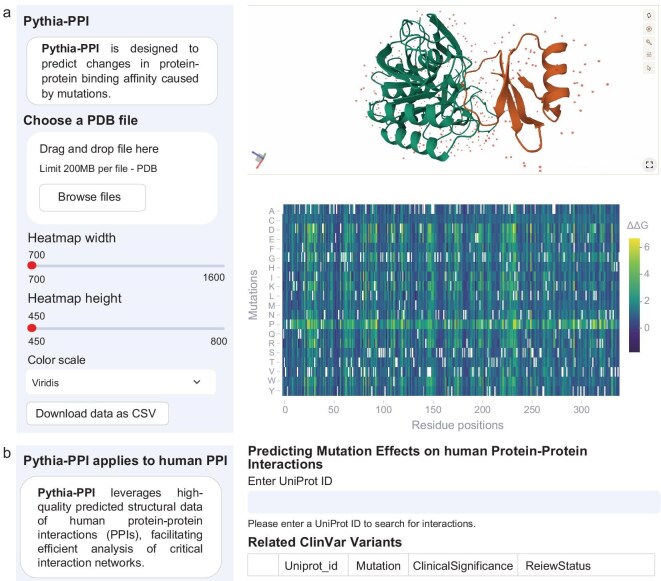
The web server of Pythia-PPI. (a) Screenshot of the Pythia-PPI platform showing the main interface for mutation analysis. The panel includes file upload options, customization settings for heat maps and a visualization of ΔΔG predictions through a heat map and an interactive 3D structure viewer. (b) Interface of the human PPI analysis module. Users can input UniProt IDs to query PPIs and analyse mutation-induced binding changes, with outputs linked to ClinVar data for clinical insights.

To enhance the accessibility of Pythia-PPI, we have developed an online web server (https://pythiappi.wulab.xyz) that enables the prediction of mutation-induced changes in binding affinity. Users can upload PDB files and generate ΔΔG predictions for all possible mutations at each residue position, which are displayed in interactive heat maps and sortable data tables. The platform allows the customization of parameters such as heat-map resolution, color scale and mutation filtering to meet diverse research needs (Fig. 6a). A specialized module for human PPIs [33] integrates a curated interaction database [34], enabling researchers to query UniProt-based protein interactions, analyse mutation-induced binding changes and link predictions to known clinical variants by using ClinVar data (Fig. 6b). Additionally, the platform provides an interactive 3D structure viewer to explore the spatial context of predicted mutations, providing structural insights into PPIs. By combining flexible visualization and user-friendly tools, Pythia-PPI facilitates efficient research for applications such as protein engineering and drug design.

## DISCUSSION

Amino acid substitutions can impact the binding strength of protein–protein complexes, with some mutations enhancing stability and others weakening or even disrupting these interactions. Such changes in binding strength can alter the structure and function of protein–protein complexes, potentially leading to the occurrence of diseases. Therefore, exploring the mutational effects on PPIs and achieving accurate and generalized predictions of ΔΔG values are of significant importance for protein engineering, disease treatment and drug design.

Through the integration of multitask learning and self-distillation, Pythia-PPI has significantly improved prediction accuracy. On the SKEMPI dataset, it achieved a Pearson's correlation of 0.7850, reflecting enhanced performance. In per-structure evaluations, it reached a Pearson's correlation of 0.5653, surpassing the previous state-of-the-art method (0.4778) and suggesting better generalization across various protein–protein complexes. In the R3669 dataset, Pythia-PPI demonstrated strong accuracy, with Pearson's and Spearman's correlations of 0.6050 and 0.6485, respectively. These results highlight the practical utility of Pythia-PPI in diverse applications, including antibody optimization, viral protein mutation fitness prediction and broader protein-engineering tasks.

The design of the Pythia model was rooted in physical principles to predict protein thermostability and generalized well for predicting protein–protein binding affinity. The success of Pythia-PPI highlighted the importance of designing artificial neural networks based on physical interpretations. The performance gains of Pythia-PPI can be attributed to the benefits of a multitask-learning and self-distillation strategy. By incorporating a protein-stability prediction task, we leveraged shared structural features between stability and binding affinity, enabling Pythia-PPI to capture a more comprehensive representation of protein structures and generalize across a wider range of structures. The self-distillation process further enhanced the model by using predictions from the original Pythia-PPI to augment the dataset. This expanded dataset offered a more exhaustive mutation landscape, providing a broader and more balanced distribution of examples, which substantially increased the accuracy and robustness of the model.

However, the self-distillation process introduced a minor bias in some predictions: compared with zero-shot predictions, the correlation between the predicted and experimental values decreased by ∼3% across all structures in the SKEMPI dataset. This decrease suggests that the self-distillation process may introduce specific biases due to the inherent limitations of model-generated data [35,36]. In addition to mitigating such biases through robust training strategies such as uncertainty weighting and reinforcement learning, the most effective way to develop better deep-learning models ultimately relies on high-quality real data [37,38]. For example, a recently published megascale dataset, encompassing nearly 1 million mutations across hundreds of proteins [39], has largely improved the prediction accuracy of protein stability [40]. A comparable dataset for PPIs would be invaluable for developing more robust deep-learning models.

The increasing availability of high-quality predicted structural models, including those for human and pathogen interactomes [41], offers promising new opportunities. The capability of Pythia-PPI for rapid, evolution-free inference makes it particularly well suited to predict the fitness landscape of these important PPIs to provide more insights into protein function and mechanisms. In future work, considering the limitations of Pythia-PPI in capturing long-range effects and conformational influences, we will explore the integration of global graph attention mechanisms and physical energy landscape information to more effectively predict both single-point and multiple-point mutation effects, thereby enhancing its robustness and applicability.

## MATERIALS AND METHODS

### Dataset curation

In this study, we utilized three experimental datasets: the SKEMPI dataset, the FireProt dataset and a deep mutagenesis dataset focused on the spike protein of SARS-CoV-2 in complex with human ACE2. The SKEMPI dataset, which contains PPI binding affinity data, and the FireProt dataset, which includes protein-stability data, were used as training sets for the model. The deep mutagenesis dataset was reserved as a blind test that was set to evaluate the generalization ability of the model. After removing incomplete and redundant entries, the SKEMPI dataset was refined to 4076 single-point mutations from 314 protein-complex structures. The FireProt dataset was curated to include 3436 mutations across 100 proteins. For the deep mutagenesis dataset, mutations not mapped to the PDB structure 6M0J were excluded, resulting in the final R3669 test set.

### Evaluation metrics

We utilized five metrics to assess the overall performance of Pythia-PPI, which include the Pearson's correlation (Pearson's correlation coefficient, *r*), Spearman's correlation (Spearman's correlation coefficient, *ρ*), RMSE, MAE and AUROC. For datasets with multiple complexes, mutation samples were grouped by structure and groups with <10 mutations were discarded. Pearson's and Spearman's correlations were then averaged across groups to obtain per-structure metrics.

### Model training

Our basic programming language is Python 3.10.13, with PyTorch 2.1.2 and a default random seed of 2024. Unless otherwise specified, Pythia-PPI is trained by using the Adam optimizer (learning rate: 1e-4; epochs: 100) and absolute error loss. We use ReduceLROnPlateau (patience: 5; decay: 0.1) to adjust the learning rate and apply early stopping (patience: 10) to prevent overfitting. All knowledge-transfer experiments use the pretrained ‘pythia-p.pt’ model on protein complexes. The batch size is 32 and all batches are sampled each epoch. The best model is selected from the training fold with the highest diversity of protein–protein complexes.

In the Pythia-PPI model, which handles two distinct tasks, the loss functions are defined separately for each task. The loss function for the PPI binding affinity prediction task is denoted as *L*_affinity_ and the loss function for the protein-stability prediction task is denoted as *L*_stability_. The overall loss function is expressed as:


\begin{eqnarray*}
{\mathrm{L}} = {\mathrm{\alpha }}{{\mathrm{L}}}_{{\mathrm{affinity}}} + \left( {1 - {\mathrm{\alpha }}} \right){{\mathrm{L}}}_{{\mathrm{stability}}}.
\end{eqnarray*}

### Baseline methods

We benchmarked Pythia-PPI against seven representative ΔΔG prediction tools: Rosetta, FoldX, ESM-1v, ESM3, MSA Transformer, DDGPred and RDE-Net. These tools span diverse modeling strategies, including physics-based models (Rosetta, FoldX), protein-language models (ESM-1v, ESM3, MSA Transformer) and deep-learning approaches (DDGPred, RDE-Net), providing a comprehensive baseline for evaluating model performance.

### Experimental validation

To validate the predictive capability of Pythia-PPI, we applied it to optimize the CB6 antibody (PDB ID: 7C01) targeting the SARS-CoV-2 PT RBD. Around 2000 single-point mutations in the heavy chain were screened and the top 10 predicted mutations were synthesized. Binding affinities were assessed by using SPR on a BIAcore 8K system. Antibodies were captured on a Protein A chip and serially diluted RBD was flowed over the surface. Kinetic data were analysed by using a 1:1 Langmuir model to calculate dissociation constants.

## Supplementary Material

nwaf231_Supplemental_File
