# Supplementary material for: Reliable prediction of protein–protein binding affinity changes upon mutations with Pythia-PPI
Source: Natl Sci Rev. 2025 Jun 10;12(6):nwaf231. doi: 10.1093/nsr/nwaf231 (PMC12199698; doi:10.1093/nsr/nwaf231)
Supplement: nwaf231_Supplemental_File [file nwaf231_supplemental_file.docx]

Reliable prediction of protein-protein binding affinity changes upon mutations with Pythia-PPI

Table of Contents

[Supplementary Figures 2](#_Toc199338213)

[Supplementary Tables 8](#_Toc199338214)

[Supplementary Methods 12](#_Toc199338215)

[1. Dataset curation 12](#_Toc199338216)

[2. Dataset splitting 12](#_Toc199338217)

[3. Evaluation metrics 13](#_Toc199338218)

[4. Model training 13](#_Toc199338219)

[5. Data augmentation 14](#_Toc199338220)

[6. Baseline Methods 14](#_Toc199338221)

[7. Enhancing CB6 antibody binding affinity using Pythia-PPI 15](#_Toc199338222)

[8. Antibody and antigen cloning 15](#_Toc199338223)

[9. DNA preparation 15](#_Toc199338224)

[10. Protein expression 16](#_Toc199338225)

[11. Antibody purification 16](#_Toc199338226)

[12. Antigen purification 16](#_Toc199338227)

[13. SPR analysis 16](#_Toc199338228)

[Supplementary Notes 18](#_Toc199338229)

[1. Generation of mutant protein-protein complex structures 18](#_Toc199338230)

[2. Details for MIF_FT 18](#_Toc199338231)

[3. Details for Pythia_FT 18](#_Toc199338232)

[4. Comprehensive performance comparison of pre-trained and fine-tuned models 19](#_Toc199338233)

[5. Detailed performance comparison with other methods on the S669 Dataset 19](#_Toc199338234)

[6. Interpretability of Pythia-PPI 21](#_Toc199338235)

[7. Analysis of prediction bias in high-impact mutations 23](#_Toc199338236)

[Reference 25](#_Toc199338237)

## Supplementary Figures


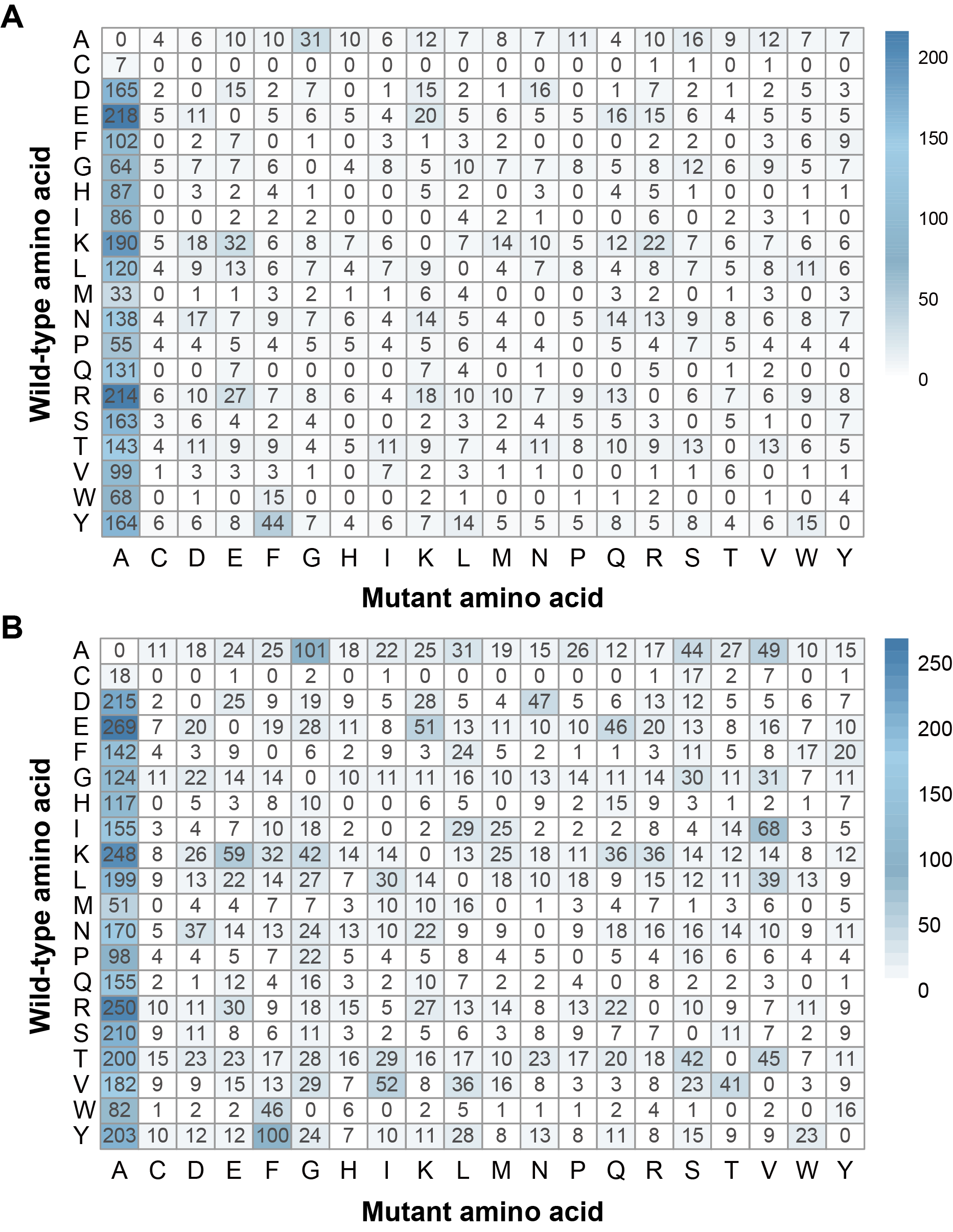


**Supplementary Figure 1.** (A) Wild-type and mutant amino acids for single-point mutations based on the SKEMPI dataset. (B) Wild-type and mutant amino acids for single-point mutations based on both the SKEMPI and the FireProt datasets.


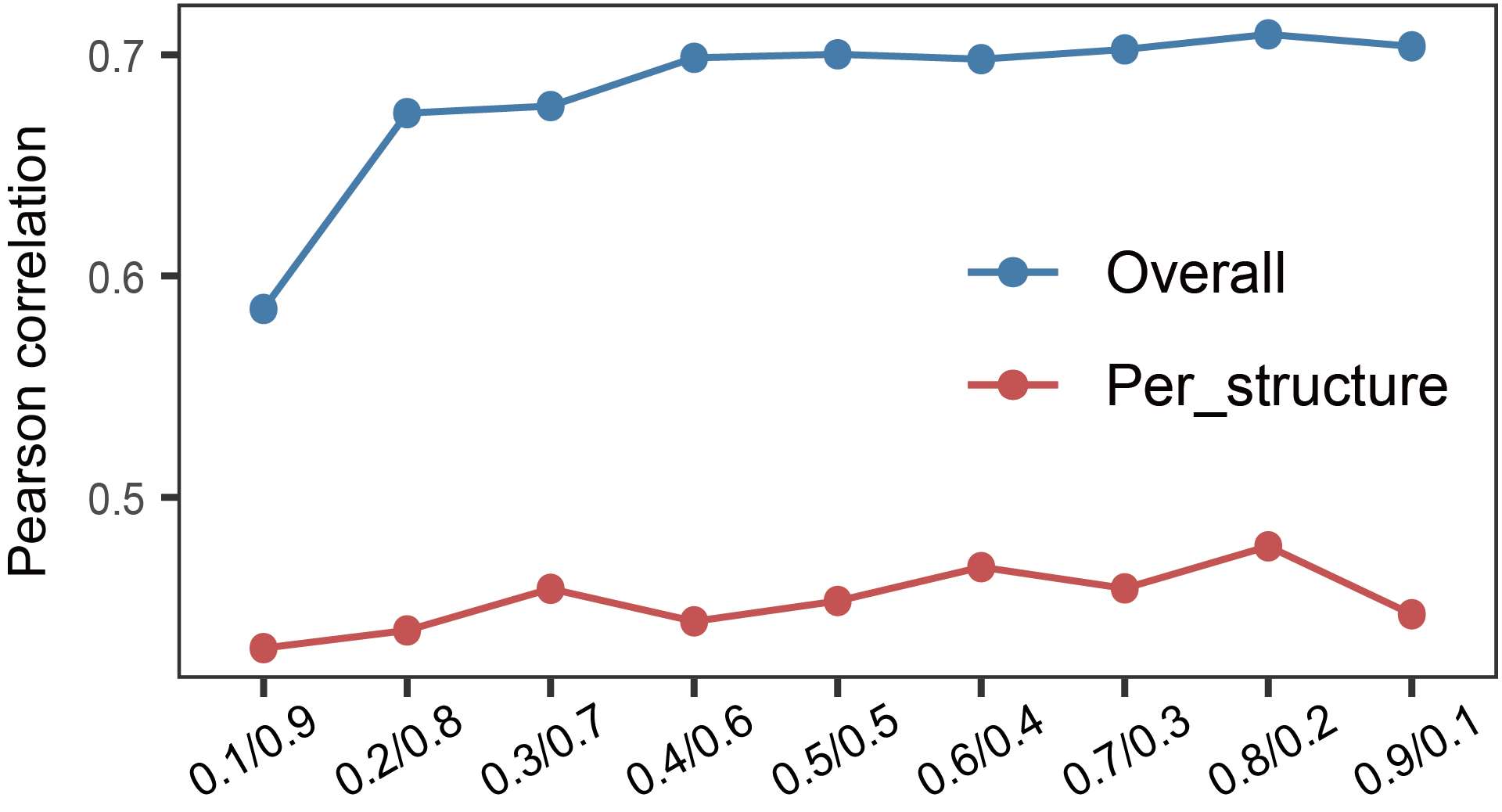


**Supplementary Figure 2.** The changes in Pearson correlation corresponding to the selection of hyperparameters for the loss functions of different tasks (in the expression α/β, α and β represent the weights of the loss function for PPI and protein ΔΔG prediction, respectively).


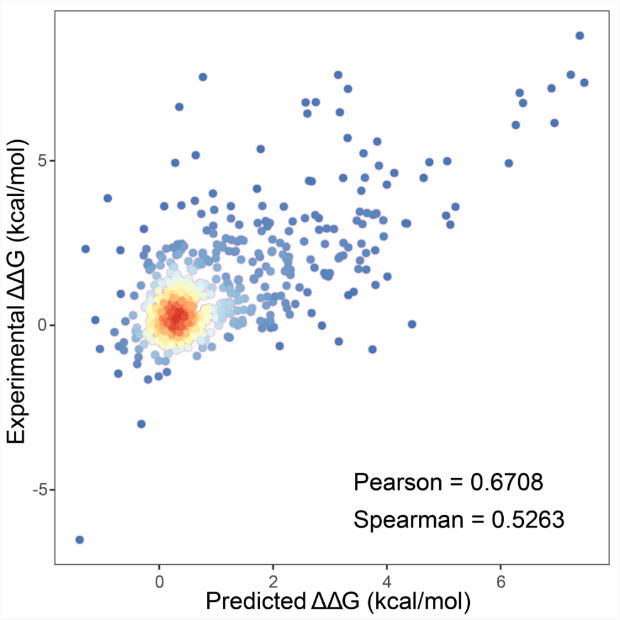


**Supplementary Figure 3.** Density scatter plot of vanilla Pythia-PPI predictions versus experimental values on the antibody-antigen subset of SKEMPI dataset.


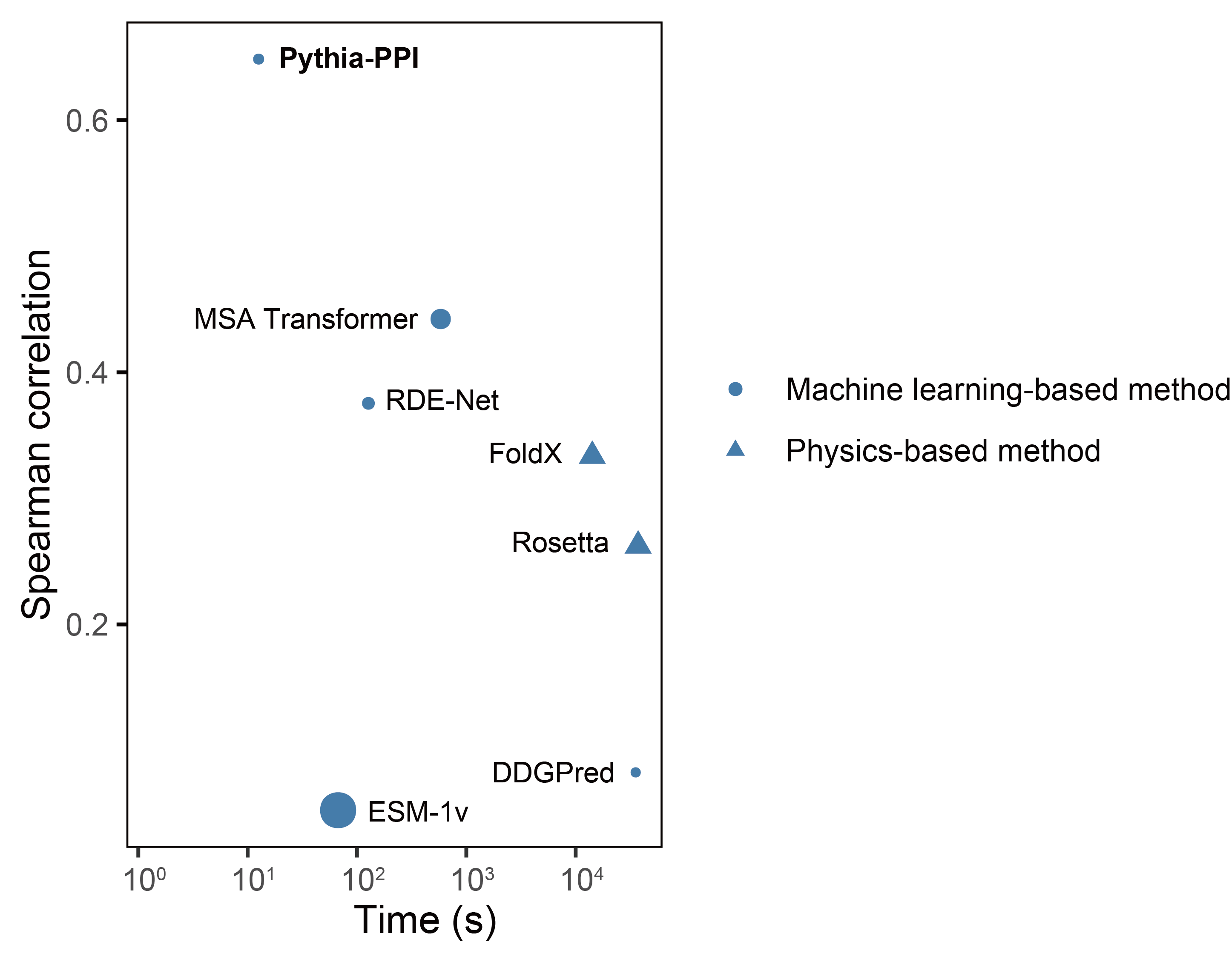


**Supplementary Figure 4.** Comparison of runtime and performance between Pythia-PPI and other methods on a protein complex (PDB ID: 6M0J), evaluated for mutation effect prediction in chain E using a single NVIDIA RTX 4090 GPU. In the figure, circles and triangles represent machine learning-based and physics-based methods, respectively. The size of each circle is proportional to the number of model parameters, with larger circles indicating methods with more parameters.


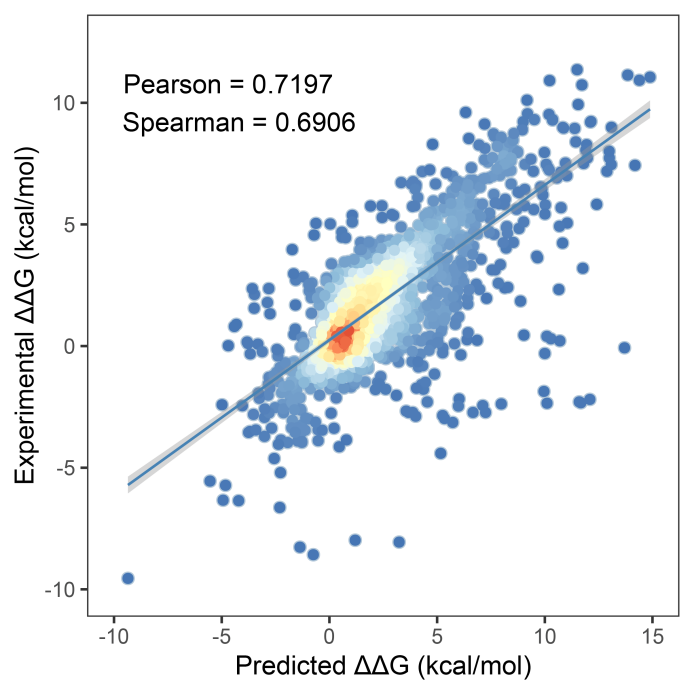


**Supplementary Figure 5.** Density scatter plot of Pythia-PPI predictions versus experimental values on the multi-mutation subset of SKEMPI dataset.


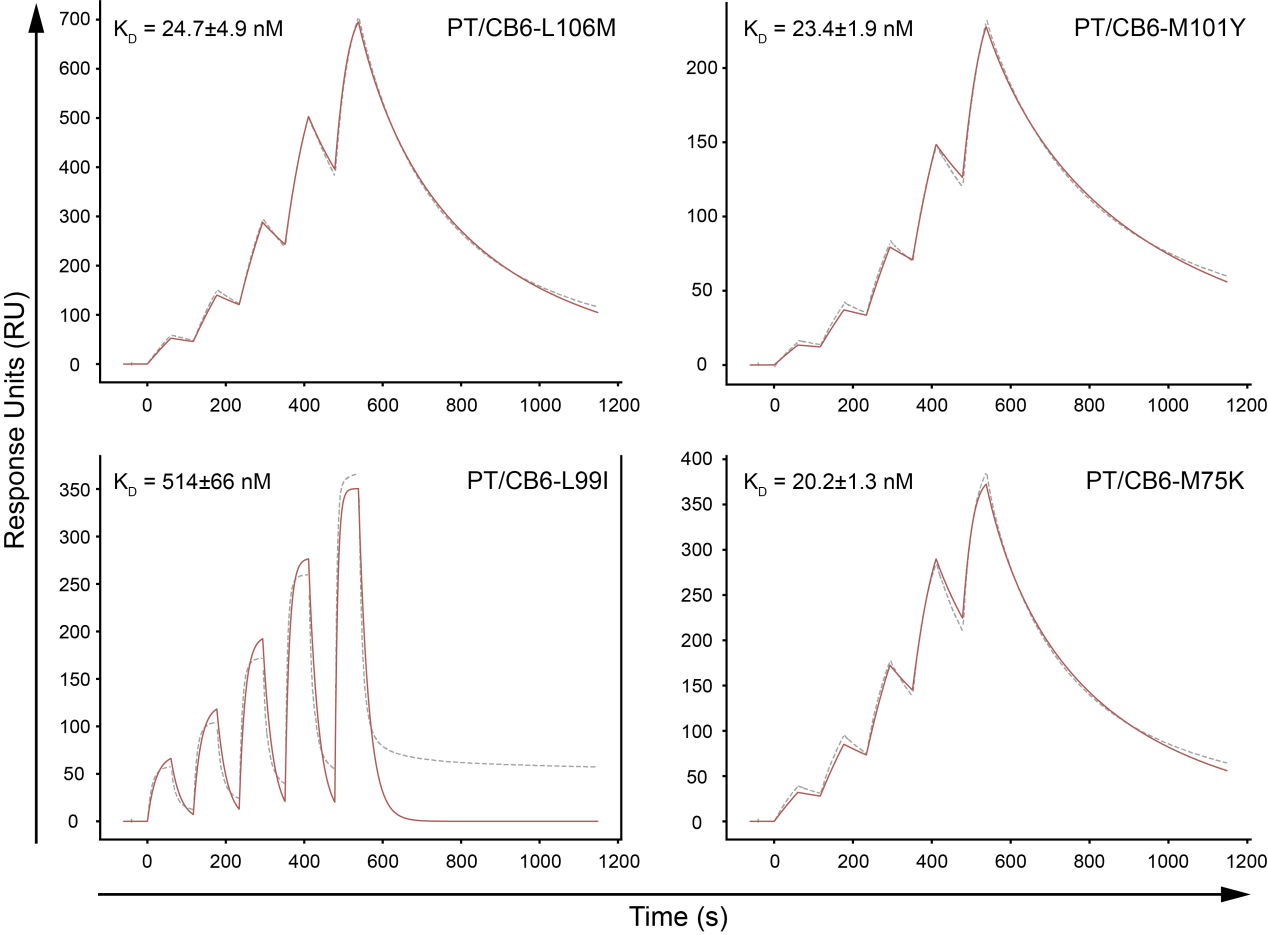


**Supplementary Figure 6.** Binding characteristics of the CB6 antibody and its mutations (CB6-L106M, CB6-M101Y, CB6-L99I, and CB6-M75K) with SARS-CoV-2 PT RBD were measured using surface plasmon resonance (SPR). The K_D_ of each is shown as the mean ± SD of three independent experiments.

## Supplementary Tables

**Supplementary Table 1.** Performance comparison of pre-trained and fine-tuned models for the SKEMPI dataset

| Method | Per-structure | | Overall | | | | |
| --- | --- | --- | --- | --- | --- | --- | --- |
|  | Pearson | Spearman | Pearson | Spearman | RMSE | MAE | AUROC |
| MIF-Δlogit | 0.2580 | 0.2364 | 0.2726 | 0.2658 | 7.7037 | 6.7320 | 0.6156 |
| Pythia-Δlogit | 0.2879 | 0.2579 | 0.3782 | 0.3036 | 5.9397 | 4.3640 | 0.6249 |
| MIF_FT | 0.4351 | 0.4059 | 0.6570 | 0.5045 | 1.3472 | 0.9067 | 0.7198 |
| Pythia_FT | 0.4462 | 0.4119 | 0.6939 | 0.5319 | 1.2687 | 0.8577 | 0.7286 |
| Vanilla Pythia-PPI | 0.4778 | 0.4486 | 0.7092 | 0.5365 | 1.2358 | 0.8496 | 0.7341 |

**Supplementary Table 2.** Runtime comparison between Pythia-PPI and other methods for predicting mutation effects in chain E of the example protein complex (PDB ID: 6M0J), evaluated on a single NVIDIA RTX 4090 GPU.

| Method | Parameters (M) | Wall-clock time (s)^*^ |
| --- | --- | --- |
| Rosetta | - | 37259.2 |
| FoldX | - | 14188.7 |
| ESM-1v | 650 | 67.2 |
| MSA Transformer | 100 | 582.5 |
| DDGPred | 1.0 | 35376.1 |
| RDE-Net | 7.2 | 127.2 |
| Pythia-PPI | 1.6 | 12.6 |

* All reported times include data preprocessing, model inference, and the generation of results from a PDB format input.

**Supplementary Table 3.** Binding data of IgG K_D_ values for CB6 antibody and its variants with SARS-CoV-2 PT RBD.

| Mutation | Avg K_D_ ± SD (nM) | Avg K_D_ Fold-Change ± SD |
| --- | --- | --- |
| WT | 14.6 ± 4.1 | NA |
| D104M | - | - |
| L106M | 24.7 ± 4.9 | 0.69 ± 0.15 |
| S31R | 8.3 ± 3.3 | 2.11 ± 0.02 |
| V63Y | 12.9 ± 1.1 | 1.21 ± 0.10 |
| S35A | 13.5 ± 0.4 | 1.14 ± 0.04 |
| M101Y | 23.4 ± 1.9 | 0.66 ± 0.04 |
| F79Y | 14.9 ± 1.0 | 1.04 ± 0.04 |
| L99I | 514 ± 66 | 0.03 ± 0.001 |
| M34W | 12.7 ± 0.8 | 1.23 ± 0.06 |
| M75K | 20.2 ± 1.3 | 0.77 ± 0.04 |

**Supplementary Table 4.** Comparison with various methods on the multi-mutation subset of SKEMPI dataset.

| Method | Per-structure | | Overall | | | | |
| --- | --- | --- | --- | --- | --- | --- | --- |
|  | Pearson | Spearman | Pearson | Spearman | RMSE | MAE | AUROC |
| Rosetta | 0.1915 | 0.0836 | 0.1991 | 0.2303 | 2.6581 | 2.0246 | 0.6207 |
| FoldX | 0.2801 | 0.2771 | 0.2347 | 0.4137 | 2.5290 | 1.8639 | 0.6828 |
| ESM-1v | -0.0599 | -0.1284 | 0.1923 | 0.1749 | 2.7586 | 2.1193 | 0.5415 |
| MSA Transformer | -0.0097 | -0.0400 | 0.0067 | 0.0030 | 2.8115 | 2.1591 | 0.4870 |
| DDGPred | 0.3912 | 0.3896 | 0.5938 | 0.5150 | 2.1813 | 1.6699 | 0.7590 |
| RDE-Net | 0.4233 | 0.3926 | 0.6288 | 0.5900 | 2.0980 | 1.5747 | 0.7749 |
| Pythia-PPI | 0.5327 | 0.5455 | 0.7197 | 0.6906 | 2.2667 | 1.4528 | 0.8080 |

## Supplementary Methods

### Dataset curation

In this study, we utilized three experimental datasets: the SKEMPI dataset, the FireProt dataset, and a deep mutagenesis dataset focused on the spike protein of severe acute respiratory syndrome coronavirus 2 (SARS-CoV-2) in complex with human angiotensin-converting enzyme 2 (ACE2). The SKEMPI dataset, which contains protein-protein interaction binding affinity data, and the FireProt dataset, which includes protein stability data, were used as training sets for the model. The deep mutagenesis dataset served as a blind test set to evaluate the model’s ability to generalize and predict mutation-induced changes in binding affinity.

Comprehensive data cleaning was applied to the SKEMPI, FireProt, and deep mutagenesis datasets to ensure data reliability. The SKEMPI dataset contains a total of 7,085 entries, including both single-point and multi-point mutations sourced from various repositories such as SKEMPI 1.0 (1), AB-Bind (2), PROXiMATE (3), and dbMPIKT (4). This dataset was carefully curated, focusing on single-point mutation samples and excluding entries with incomplete, uncertain, or unreliable data. Calculations were then performed to determine the average change in binding affinity for identical single-point mutations across the same protein-protein complexes. As a result, the SKEMPI dataset was refined to include 4,076 single-point mutations across 314 complexes. Mutations in the SARS-CoV-2 spike protein not present in the reference PDB structure (6M0J), specifically those in the first two N-terminal and last five C-terminal residues, were excluded, resulting in the filtered R3669 dataset. Following the cleaning principles established by Dieckhaus et al. (5), the final filtered FireProt dataset contains 3,436 single-point mutations from 100 proteins.

### Dataset splitting

To ensure a robust and comprehensive assessment of the model’s performance, a protein-level 5-fold cross-validation strategy was implemented. Specifically, the SKEMPI dataset was divided into five folds based on protein complex structures. In each iteration, onefold was designated as the validation set, while the remaining folds formed the training set. This cross-validation procedure was repeated five times, each time with a different fold used as the validation set, thereby ensuring a thorough and accurate evaluation. The average performance metrics from these five iterations were then used as the final evaluation metric for the model. Additionally, the FireProt dataset, was entirely used for model training to enhance the model's performance.

The distribution of mutation samples across the five folds in the SKEMPI dataset was as follows: the first fold included 841 mutations from 75 protein-protein complexes; the second fold contained 819 mutations from 56 complexes; the third fold comprised 980 mutations from 53 complexes; the fourth fold included 819 mutations from 91 complexes; and the fifth fold encompassed 617 mutations from 39 complexes.

### Evaluation metrics

We utilized five metrics to assess the overall performance of the model, which include the Pearson correlation (Pearson correlation coefficient, r), Spearman correlation (Spearman correlation coefficient, ρ), RMSE (Root Mean Square Error), MAE (Mean Absolute Error), and AUROC (Area Under the Receiver Operating Characteristic Curve).

The formulas for these metrics are as follows:

$$r=\frac{\sum_{i=1}^{n} (x_{i}-\bar{x})(y_{i}-\bar{y})}{\sqrt{\sum_{i=1}^{n} (}x_{i}-\bar{x})^{2}\sqrt{\sum_{i=1}^{n} (}y_{i}-\bar{y})^{2}}$$

$$\rho=\frac{\sum_{i=1}^{n} (r_{i}-\bar{r})(s_{i}-\bar{s})}{\sqrt{\sum_{i=1}^{n} (}r_{i}-\bar{r})^{2}\sqrt{\sum_{i=1}^{n} (}s_{i}-\bar{s})^{2}}$$

$$RMSE=\sqrt{\frac{1}{n}\sum_{i=1}^{n} (y_{i}-\hat{y}_{i})^{2}}$$

$$\text{MAE}=\frac{1}{n}\sum_{i=1}^{n} |y_{i}-\hat{y}_{i}|$$

These metrics were employed to analyze the relationship between the model's predicted ΔΔG values and experimentally determined ΔΔG values. In practical applications, particular attention is often given to the correlation for individual protein-protein complexes. Consequently, for datasets containing multiple complexes, we grouped the mutation samples by structure following principles established by Luo et al. (6). Groups containing fewer than ten mutation samples were discarded. We then calculated the correlation for each group and averaged these results to obtain two additional metrics: the per-structure Pearson and Spearman correlations.

### Model training

Our base programming language is Python 3.10.13, on which PyTorch 2.1.2 is used, with the default random seed set to 2024. Unless otherwise specified, Pythia-PPI is trained using the Adam optimizer with a learning rate set at 1e-4 and epochs set at 100. The training loss is computed using the absolute value error loss function. We employ a callback function (ReduceLROnPlateau) to update the learning rate, with a patience setting of 5 and a decay factor of 0.1. To prevent overfitting, early stopping is implemented with a patience setting of 10. All knowledge transfer experiments utilize the 'pythia-p.pt' model from Pythia, which the pretraining stage involved protein complexes. The batch size is set at 32, and all batches are sampled in each epoch. At the end of training, the model from the training fold with the highest diversity of protein-protein complexes is selected as the best model.

In the Pythia-PPI model, which handles two distinct tasks, the loss functions are defined separately for each task. The loss function for the PPI binding affinity prediction task is denoted as Laffinity, and the loss function for the protein stability prediction task is denoted as Lstability. The overall loss function is expressed as:

$$L=\alpha L_{\mathrm{affinity}}+(1-\alpha)L_{\mathrm{stability}}$$

Training the Pythia-PPI model on the SKEMPI and FireProt datasets typically requires about half an hour on a single V100 GPU, supported by 10 dedicated CPUs.

### Data augmentation

To create the augmented dataset, we selected residues located on the protein-protein interface across all structures in the SKEMPI dataset. Residue pairs located on different chains and with the Cα distance within 8Å were identified as interface residues. The ∆∆G values of all possible mutations were predicted using vanilla Pythia-PPI as augmented data, which was then used to train the final Pythia-PPI model, following the same settings as those used for training vanilla Pythia-PPI.

### Baseline Methods

We utilized six different tools to predict ΔΔG values on the R3669 dataset, using the default model weights and inference processes for all methods. Here is a brief overview of their characteristics:

Rosetta (7) is a physics-based method that evaluates the stability of protein structures using an energy function. It combines Monte Carlo sampling algorithms to explore structural space and fragment assembly techniques to accurately predict and design protein structures.

FoldX (8) is another physics-based method that predicts protein structure and stability by calculating the interaction energy between individual atoms within the protein and between different amino acids.

ESM-1v (9) is a machine learning-based language model capable of zero-shot prediction, which allows it to predict the impact of protein mutations on function without specific training data.

ESM 3 (10) is an advanced machine learning-based multimodal protein model that integrates sequence, structure, and functional information, using a bidirectional Transformer architecture and geometric attention mechanisms to capture complex interactions among these multimodal data.

MSA Transformer (11) is a machine learning-based language model that uses the self-attention mechanisms of Transformers to capture dependencies between sequences. It enhances protein sequence representation by modeling multiple sequence alignment data.

DDGPred (12) is a supervised machine learning method that uses an attention-based geometric neural network structure to learn the geometric effects of protein-protein interactions from three-dimensional protein-protein complex structures.

RDE-Net (6) is also a machine learning method that utilizes unsupervised learning with Rotamer Density Estimation (RDE) by simulating the conformations of side-chain atoms to capture atomic interactions and learn rotameric density representations.

These tools provide a comprehensive suite of methods for assessing and predicting the effects of mutations in PPI, leveraging both traditional physics-based techniques and cutting-edge machine learning-based models.

### Enhancing CB6 antibody binding affinity using Pythia-PPI

We selected PDB 7C01 for improving the binding affinity of the CB6 antibody in complex with SARS-CoV-2 PT RBD. This antibody, originally isolated from convalescent COVID-19 patients, binds to the RBD of the SARS-CoV-2 spike protein, thereby blocking its interaction with the ACE2 receptor on host cells and effectively preventing viral infection. To optimize its binding affinity, we utilized Pythia-PPI to predict the effects of approximately 2,000 single-point mutations in the heavy chain variable region. Based on these predictions, we identified the top 10 high-affinity mutations at distinct residues. These selected mutations were synthesized and further validated through experimental testing.

### Antibody and antigen cloning

Based on the heavy and light chain sequences of the CB6 antibody published in the NCBI database, the heavy chain was fused to the constant region of human IgG1, while the light chain was fused to the Igκ chain. The CB6 wild-type heavy and light chains were constructed by cloning them into the pCAGGS vector, respectively. According to the mutation sites predicted by the model, single-point mutations were introduced into the heavy chain and cloned into the pCAGGS vector in the same manner. The SARS-CoV-2 prototype RBD (residues 319-541, GISAID: EPI_ISL_402119) was cloned into the pCAGGS vector with a 6 × His tag at the C-terminus to facilitate protein purification.

### DNA preparation

The plasmid was transformed into *E. coli* DH5α competent cells. The transformed cells were evenly spread onto culture plates containing ampicillin and incubated at 37°C for 16 hours. A single clone was picked and inoculated into 500 μL of LB medium. After 6-8 hours of incubation, the bacterial culture was transferred into 300 mL of LB medium and further incubated for 16 hours. Bulk plasmid preparation was performed using the Endotoxin-Free Plasmid Bulk Kit (TIANGEN). The prepared plasmids were stored at 4°C.

### Protein expression

Antibodies and antigens were expressed in HEK293F cells. HEK293F cells were cultured in SMM 293-TII Expression Medium (Sino Biological) and maintained at 37°C with 5% CO₂. The day before transfection, the cells were counted and passaged to a density of 1-1.5 × 10⁶ cells/mL. On the following day, transfections were performed when the cell density reached 2-3 × 10⁶ cells/mL. For antibody transfection, the heavy chain and light chain plasmids were co-transfected into HEK293F cells at a 2:3 ratio. Plasmids and PEI were mixed and transfected at a 1:3 ratio. Five days post-transfection, the supernatant was collected, centrifuged at 8,000 rpm and 8°C for 1 hour, and then filtered through a 0.22 μM filter.

### Antibody purification

Antibodies were purified using Protein A 5 mL affinity columns (GE Healthcare) and Superdex^TM^ 200 10/300 GL columns (GE Healthcare). 20 mM Na_3_PO^4^, pH 7.4 was used as buffer A and 100 mM Glycine, pH 3.0 was used as buffer B. The Protein A column was eluted with buffer A and buffer B. Heteroproteins were first washed out of the column with buffer A, and then the antibody proteins were eluted with buffer B. The antibody proteins obtained were concentrated and then further purified using a Superdex^TM^ 200 10/300 GL column pre-equilibrated with PBS buffer.

### Antigen purification

Antigens were purified using HisTrap HP 5 mL affinity columns (GE Healthcare) followed by Superdex^TM^ 200 10/300 GL columns (GE Healthcare). PBS (10 mM Na₂HPO₄, 2 mM KH₂PO₄, 137 mM NaCl, 2.7 mM KCl, pH 7.4) was used as buffer A, while PBS supplemented with 1M imidazole (pH 7.5) was used as buffer B. The HisTrap column was eluted using a gradient of buffer B with concentrations of 0 mM, 20 mM, 50 mM, 100 mM, 300 mM, and 1000 mM. Based on SDS-PAGE analysis, proteins eluted at 100 mM or 300 mM were collected and concentrated. Further purification was carried out using a Superdex^TM^ 200 10/300 GL column pre-equilibrated with PBS buffer.

### SPR analysis

The binding affinity between the RBD and the antibody was determined and analyzed using the BIAcore 8K system (GE Healthcare). PBST (PBS + 0.05% (v/v) Tween 20) was used as the running buffer. Protein A chip (GE Healthcare) was used to capture the antibody, and the response units (RU) for antibody capture were adjusted to approximately 1000 RU by modifying the antibody concentration. Serial dilutions of RBD were then flowed over the chip surface to measure binding affinity. The chip was regenerated using 10 mM Glycine, pH 1.7. Data were analyzed using BIAcore 8K evaluation software, and the equilibrium dissociation constants (K_D_) for each interaction pair were calculated using a 1:1 (Langmuir) binding model.

## Supplementary Notes

### Generation of mutant protein-protein complex structures

To investigate the mutant structures of the R3669 dataset (PDB ID: 6M0J) and the SKEMPI dataset, we employed the comparative modeling software MODELLER (13). Utilizing the wild-type structure as a template and adhering to the default parameters of the software, we constructed models of the mutant protein-protein complexes. During this process, MODELLER reconstructed the side chains of the mutated residues and made minor adjustments to the backbone and side chains of the entire complex. These modifications helped eliminate spatial conflicts and optimized the atomic interactions with the new mutant residues.

### Details for MIF_FT

The MIF initially encodes amino acid sequences as node features in a graph. Subsequently, it employs a Graph Convolutional Network (GCN) to perform convolution operations on these node features, aiming to capture relational information among the nodes. Following this, MIF utilizes known protein structures as input, converting structural information into graph edge features. The GCN is then reapplied to these edge features to extract interaction information between residues. The MIF model is pretrained to obtain feature representations, and a multilayer perceptron is integrated to adapt these feature inputs. It is important to note that the amino acid sequence generation code embedded within MIF is solely designed for monomeric proteins. Modifications are required to adapt this code for generating sequences of protein complexes.

### Details for Pythia_FT

Pythia is an autonomous graph neural network that integrates attention mechanisms with message passing neural networks (MPNN), focusing on identifying critical substructures during the learning process. These substructures are essential for understanding interaction properties. In Pythia, each amino acid is treated as a node, connected based on the Euclidean distance to the nearest 32 amino acids from the C-α atom. The model utilizes one-hot encoding to represent each amino acid type and employs sine and cosine functions to express main-chain dihedral angles (φ, ψ, and ω) as node input features. For edge features, Pythia considers the distances between five main-chain atoms (C-α, C, N, O, and C-β), along with their sequence positions and chain information. Through pretraining the Pythia model, we obtain feature representations and incorporate a multilayer perceptron to adapt these feature inputs. It is important to note that extracting corresponding wild-type and mutant features based on amino acid types and mutation positions is essential for feature extraction.

### Comprehensive performance comparison of pre-trained and fine-tuned models

We employed seven evaluation metrics to comprehensively compare the performance of MIF-Δlogit, Pythia-Δlogit, MIF_FT, Pythia_FT, and vanilla Pythia-PPI. The observations indicate that, compared to the pre-trained models, the fine-tuned models achieved an improvement of over 80% in overall Pearson correlation and more than 50% in per-structure correlation. Furthermore, both fine-tuned Pythia_FT compared to MIF_FT and Pythia-Δlogit relative to MIF-Δlogit outperformed across all evaluation metrics, demonstrating that representations derived from Pythia are indeed superior to those from MIF. Additionally, vanilla Pythia-PPI, built upon the foundation of Pythia_FT, further enhances performance comprehensively, particularly notable in terms of per-structure correlation.

### Detailed performance comparison with other methods on the S669 Dataset

Pythia-PPI consistently demonstrates superior performance across both the direct mutation set S669-DIR and the reverse mutation set S669-REV (14). As shown in Table S5, for S669-DIR, Pythia-PPI achieves a Pearson correlation of 0.4782 and a Spearman correlation of 0.4975, indicating strong agreement between predicted and experimental ΔΔG values. Its RMSE and MAE values are 1.5200 and 1.1000, respectively, reflecting high predictive accuracy and low error. In S669-REV, Pythia-PPI maintains robust performance with a Pearson correlation of 0.4743 and a Spearman correlation of 0.4679. The RMSE and MAE values for these reverse mutations are 1.8201 and 1.3885, respectively, showing slightly higher error but still strong predictive capability. Compared to other methods, such as MAESTRO, which performs well in S669-DIR but drops significantly in S669-REV, Pythia-PPI demonstrates consistent reliability. The consistent performance of Pythia-PPI across various evaluation metrics underscores its effectiveness in capturing the impacts of point mutations on protein-protein interactions.

**Supplementary Table 5.** Performance comparison of fourteen methods for the S669 dataset

| Method | S669-DIR | | | | S669-REV | | | | |
| --- | --- | --- | --- | --- | --- | --- | --- | --- | --- |
|  | Pearson | Spearman | RMSE | MAE | | Pearson | Spearman | RMSE | MAE |
| Pythia-PPI | 0.4782 | 0.4975 | 1.5200 | 1.1000 | | 0.4743 | 0.4679 | 1.8201 | 1.3885 |
| MAESTRO (15) | 0.4967 | 0.4634 | 1.4444 | 1.0619 | | 0.1977 | 0.1931 | 2.0972 | 1.6464 |
| ACDC-NN (16) | 0.4597 | 0.4540 | 1.4884 | 1.0454 | | 0.4505 | 0.4422 | 1.5048 | 1.0560 |
| DDGun3D (17) | 0.4324 | 0.4271 | 1.5971 | 1.1091 | | 0.4105 | 0.4106 | 1.6184 | 1.1438 |
| INPS3D (18) | 0.4303 | 0.4390 | 1.5028 | 1.0683 | | 0.3279 | 0.3585 | 1.7647 | 1.3083 |
| Pythia | 0.4292 | 0.4637 | 8.3886 | 6.1554 | | 0.4331 | 0.4453 | 6.3759 | 4.7321 |
| Dynamut (19) | 0.4148 | 0.3737 | 1.5955 | 1.1851 | | 0.3444 | 0.3649 | 1.6948 | 1.2378 |
| PoPMuSiC (20) | 0.4146 | 0.4149 | 1.5138 | 1.0879 | | 0.2395 | 0.2189 | 2.0856 | 1.6403 |
| DUET (21) | 0.4133 | 0.4160 | 1.5225 | 1.0962 | | 0.2283 | 0.2312 | 2.1412 | 1.6808 |
| SDM (22) | 0.4105 | 0.3920 | 1.6706 | 1.2644 | | 0.1348 | 0.1377 | 2.1562 | 1.6368 |
| PremPS (23) | 0.4050 | 0.4165 | 1.5128 | 1.0924 | | 0.4191 | 0.4210 | 1.4868 | 1.0541 |
| ThermoNet (24) | 0.3911 | 0.3733 | 1.6170 | 1.1742 | | 0.3785 | 0.3381 | 1.6573 | 1.2349 |
| mCSM (25) | 0.3593 | 0.3628 | 1.5438 | 1.1296 | | 0.2202 | 0.2086 | 2.3007 | 1.8598 |
| FoldX | 0.2141 | 0.2827 | 2.3179 | 1.5683 | | 0.2175 | 0.3390 | 2.4761 | 1.500 |
| I-Mutant3.0 (26) | 0.3589 | 0.3540 | 1.5366 | 1.1245 | | 0.1512 | 0.1537 | 2.3181 | 1.8678 |

### Interpretability of Pythia-PPI

To better explain Pythia-PPI's accuracy in predicting mutation effects, we conducted an analysis based on structural mechanisms and attention maps. Using the antibody HyHEL-63 Y33I mutation in complex with hen egg-white lysozyme (HEL) (PDB ID: 1XGR) as an example (27), we explored the model's performance in predicting three different types of mutations: neutral mutation I33L, affinity-increasing mutation I33F, and affinity-decreasing mutation I33A. From a structural perspective, in combination with the mechanistic analysis by Li et al. (27), when I33 is replaced by L33, the structural similarity between L33 and I33 leads to no significant effect on protein-protein interactions. However, when I33 is replaced by F33, F33 enhances the interaction with HEL K97 and other key residues (such as D32, S52, Y53, W98), thereby improving binding affinity. In contrast, when I33 is replaced by A33, the shorter side chain of alanine causes the loss of interaction with HEL K97 and van der Waals contacts with other critical residues, resulting in a decreased binding affinity (Figure S7A). Additionally, attention map analysis reveals that Pythia-PPI, by learning from the local structure of protein complexes, effectively identifies and learns key amino acid residues that influence protein interactions (Figure S7B). Although the attention scores for these residues are not particularly high, the model's high accuracy in predicting the effects of the three mutations suggests that Pythia-PPI enhances its prediction capabilities by incorporating factors such as the spatial positioning of local structures and the chemical properties of amino acids (Figure S7A).

Furthermore, in practical applications, we are generally more concerned with mutations that have a significant impact on protein-protein interactions. The energetics of protein-protein binding reactions are primarily governed by hot spot residues, which almost always cluster at the interface, with relatively fewer such residues located at the edge (27). To better understand Pythia-PPI's performance in predicting high-impact mutations, we focused on analyzing mutated residues at the interface, defined as those with a Cα atom distance of less than 5 Å from interacting chain residues. As shown in Figure S7C, when predicting changes in binding affinity, Pythia-PPI predominantly focuses on residues from the interacting chain rather than those from the mutated chain. This observation suggests that Pythia-PPI actively attends to residues that have a substantial effect on the interaction. To further illustrate this, we selected the trypsin-BPTI complex (PDB ID: 2FTL) as a case study (28). In this complex, the K15 side chain from chain I forms a strong electrostatic interaction with D175 from chain E (29, 30). As shown in Figure S7D, Pythia-PPI assigns higher attention to residues in chain E, indicating that Pythia-PPI is capable of learning the binding environment of the interacting protein more effectively. Moreover, studies have shown that when BPTI K15 is mutated to aromatic residues (W, F), large hydrophobic residues (M, L), and polar residues (Q, N, S, H), it can generally form more stable complexes with trypsin compared to mutated to small residues (G, A), β-branched residues (T, V, I), and acidic residues (D, E) (29). As shown in Table S6, Pythia-PPI predicts higher ΔΔG values for mutation types (G, A, T, V, I, D, E) compared to other mutation types, consistent with the finding. These results further validate the robustness of Pythia-PPI, demonstrating its ability to accurately capture the interactions between mutated residues with different properties and their surrounding environments.


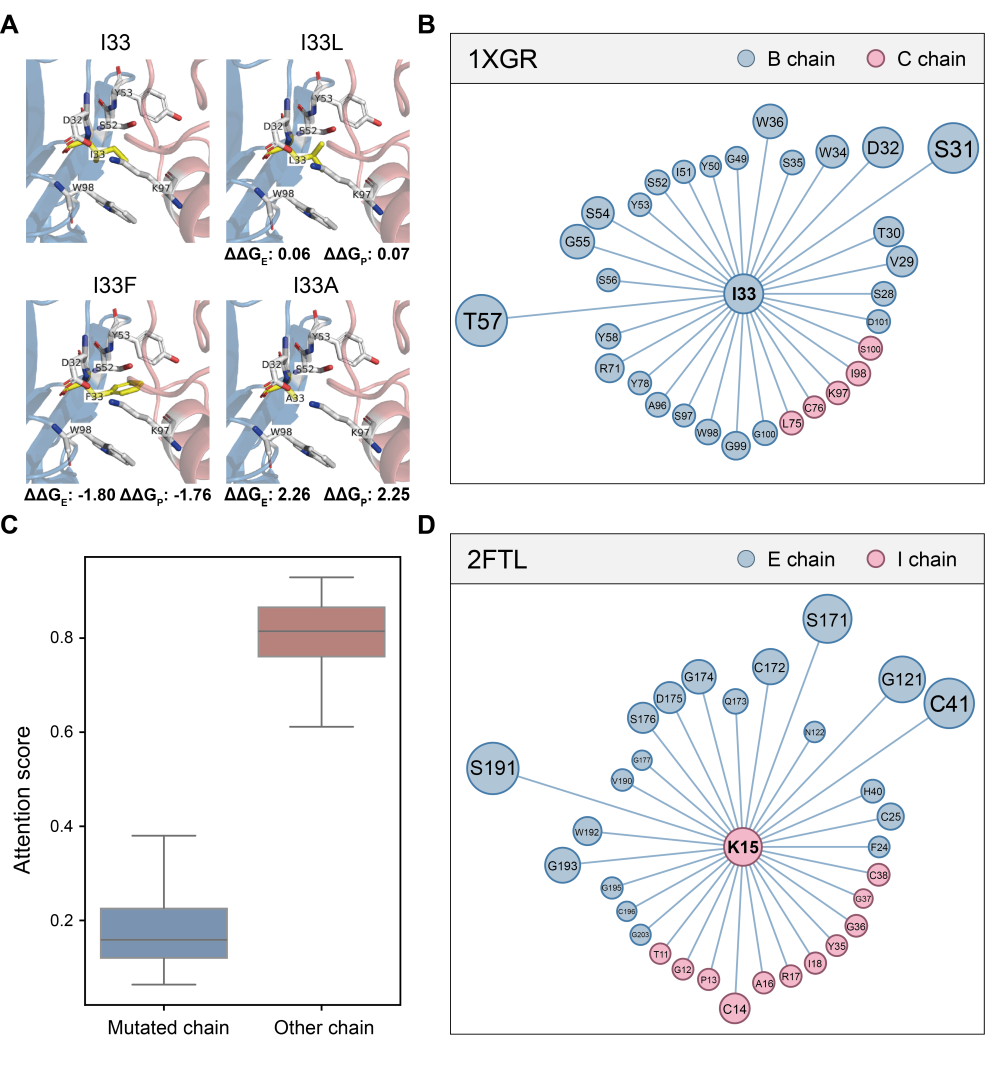


**Supplementary Figure 7.** Interpretability of Pythia-PPI. (A) Local structure of the antibody HyHEL-63 Y33I mutation in complex with hen egg-white lysozyme (HEL) (PDB ID: 1XGR), along with the local structures of HyHEL-63 I33L, I33F, and I33A. ΔΔG_E_ denotes the experimental ΔΔG, and ΔΔG_P_ denotes the predicted ΔΔG. (B) In the k-nearest neighbors (k-NN) graph of I33 in the HyHEL-63, the weights are assigned based on the attention score in the final attention message-passing layer (AMPL). (C) Boxplot showing the distribution of attention scores assigned by interface residues to residues on the mutated chain and other interacting chain in the SKEMPI dataset. Interface residues are defined as those whose Cα atoms are within 5 Å of residues on the interacting chain. (D) In the k-nearest neighbors (k-NN) graph of K15 in the BPTI, the weights are assigned based on the attention score in the final attention message-passing layer (AMPL).

### Analysis of prediction bias in high-impact mutations

To investigate the wide range of ΔΔG values predicted by Pythia-PPI for certain high-impact mutations, we selected mutation samples from the SKEMPI dataset with experimental ΔΔG values greater than 5 kcal/mol in absolute value for analysis (Figure S8). For mutations that significantly enhance binding affinity (ΔΔG ≤ –5 kcal/mol), despite a certain degree of prediction bias, the predicted ΔΔG values exhibit a strong correlation with experimental ΔΔG values (Pearson = 0.78, Spearman = 0.76). However, for mutations that significantly weaken binding affinity (ΔΔG ≥ 5 kcal/mol), we observed considerable deviations between the predicted and experimental ΔΔG values in some mutation samples. For example, in the trypsin-BPTI complex (PDB ID: 2FTL), the majority of high-impact mutants have experimental K_D_ values below 10⁻⁶ M, indicating a stable binding conformation as shown in Table S6. In this case, the model's prediction errors are relatively low (ranging from 0 to 1.07 kcal/mol), and a strong correlation between experimental and predicted ΔΔG values is maintained (Pearson = 0.96, Spearman = 0.94). In contrast, in certain complexes, the prediction errors increase significantly. For instance, in the cytochrome C peroxidase-cytochrome C (PDB ID: 4JFF) and TCR-MHC (PDB ID: 2PCC), the MAE of the model reaches as high as 5.66 kcal/mol. as depicted in Table S6, the K_D_ values of these mutants range from 10⁻⁵ to 10⁻² M, reflecting weak or unstable binding states. In such cases, the static crystal structures may fail to accurately capture the actual binding conformation, leading the model to overestimate the binding strength. Moreover, the experimental ΔΔG values in these low-affinity systems often arise from transient and rare binding events, introducing significant uncertainty into the experimental data and further increasing prediction bias.


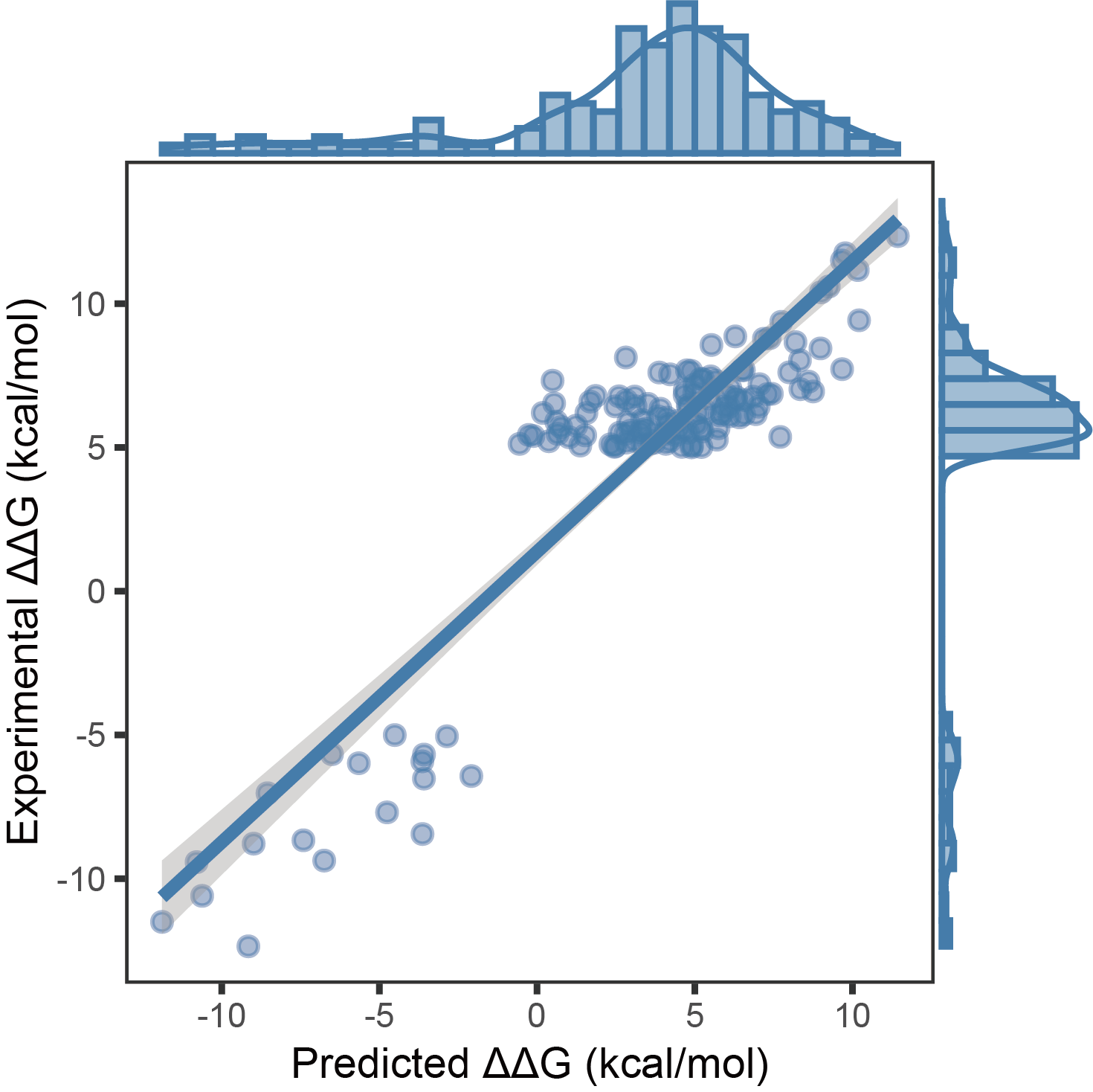


**Supplementary Figure 8.** Correlation and distribution between predicted and experimental ΔΔG values for high-impact mutations (with absolute experimental ΔΔG ≥ 5 kcal/mol) in the SKEMPI dataset.

**Supplementary Table 6.** Performance of Pythia-PPI on high-impact mutations across example protein complexes.

| PDB ID | Mutation | Experimental ΔΔG  (kcal/mol) | Predicted ΔΔG  (kcal/mol) | Absolute error  (kcal/mol) | K_D_  (M) |
| --- | --- | --- | --- | --- | --- |
| 2FTL | KI15A | 10.40 | 10.30 | 0.10 | 3.7E-06 |
|  | KI15G | 12.35 | 12.35 | 0.00 | 6.7E-05 |
|  | KI15S | 7.72 | 8.79 | 1.07 | 2.7E-08 |
|  | KI15V | 11.76 | 11.61 | 0.15 | 2.4E-05 |
|  | KI15T | 10.60 | 10.08 | 0.52 | 3.5E-06 |
|  | KI15D | 11.50 | 11.09 | 0.41 | 1.6E-05 |
|  | KI15N | 8.03 | 8.28 | 0.25 | 4.6E-08 |
|  | KI15M | 7.69 | 7.33 | 0.36 | 2.6E-08 |
|  | KI15I | 11.17 | 11.53 | 0.36 | 9.1E-06 |
|  | KI15L | 8.86 | 8.63 | 0.23 | 1.9E-07 |
|  | KI15E | 9.42 | 10.39 | 0.97 | 4.8E-07 |
|  | KI15Q | 9.37 | 9.21 | 0.16 | 4.4E-07 |
|  | KI15H | 8.78 | 8.08 | 0.70 | 1.6E-07 |
|  | KI15F | 7.03 | 7.18 | 0.15 | 8.3E-09 |
|  | KI15Y | 6.86 | 6.04 | 0.82 | 6.3E-09 |
|  | KI15W | 8.66 | 8.77 | 0.11 | 1.3E-07 |
| 4JFF | EC1A | 7.32 | 2.03 | 5.29 | 1.4E-04 |
|  | GC4A | 6.52 | 6.08 | 0.44 | 3.6E-05 |
|  | GC6A | 6.60 | 3.06 | 3.54 | 4.1E-05 |
|  | IC7A | 6.43 | 6.20 | 0.23 | 3.1E-05 |
|  | LC8A | 6.20 | 2.89 | 3.31 | 2.1E-05 |
|  | TC9A | 6.53 | 2.40 | 4.13 | 3.7E-05 |
| 2PCC | EA290A | 6.20 | 0.54 | 5.66 | 4.1E-02 |

## Reference

1. Moal,I.H. and Fernández-Recio,J. (2012) SKEMPI: a structural kinetic and energetic database of mutant protein interactions and its use in empirical models. Bioinformatics, 28, 2600–2607.
2. Sirin,S., Apgar,J.R., Bennett,E.M. and Keating,A.E. (2016) AB-Bind: antibody binding mutational database for computational affinity predictions. Protein Science, 25, 393–409.
3. Jemimah,S., Yugandhar,K. and Michael Gromiha,M. (2017) PROXiMATE: a database of mutant protein-protein complex thermodynamics and kinetics. Bioinformatics, 33, 2787–2788.
4. Liu,Q., Chen,P., Wang,B., Zhang,J. and Li,J. (2018) dbMPIKT: a database of kinetic and thermodynamic mutant protein interactions. BMC Bioinformatics, 19, 455.
5. Dieckhaus,H., Brocidiacono,M., Randolph,N.Z. and Kuhlman,B. (2024) Transfer learning to leverage larger datasets for improved prediction of protein stability changes. Proceedings of the National Academy of Sciences of the United States of America, 121, e2314853121.
6. Luo,S., Su,Y., Wu,Z., Su,C., Peng,J. and Ma,J. (2023) Rotamer density estimator is an unsupervised learner of the effect of mutations on protein-protein interaction. Proceedings of the 11th International Conference on Learning Representations.
7. Kortemme,T. and Baker,D. (2002) A simple physical model for binding energy hot spots in protein–protein complexes. Proceedings of the National Academy of Sciences of the United States of America, 99, 14116–14121.
8. Delgado,J., Radusky,L.G., Cianferoni,D. and Serrano,L. (2019) FoldX 5.0: working with RNA, small molecules and a new graphical interface. Bioinformatics, 35, 4168–4169.
9. Meier,J., Rao,R., Verkuil,R., Liu,J., Sercu,T. and Rives,A. (2021) Language models enable zero-shot prediction of the effects of mutations on protein function. Advances in Neural Information Processing Systems, 34, 29287–29303.
10. Hayes,T., Rao,R., Akin,H., Sofroniew,N.J., Oktay,D., Lin,Z., Verkuil,R., Tran,V.Q., Deaton,J., Wiggert,M. et al. (2025) Simulating 500 million years of evolution with a language model. Science, 387, 850–858.
11. Rao,R.M., Liu,J., Verkuil,R., Meier,J., Canny,J., Abbeel,P., Sercu,T. and Rives,A. (2021) MSA transformer. Proceedings of the 38th International Conference on Machine Learning, 139, 8844–8856.
12. Shan,S., Luo,S., Yang,Z., Hong,J., Su,Y., Ding,F., Fu,L., Li,C., Chen,P., Ma,J. et al. (2022) Deep learning guided optimization of human antibody against SARS-CoV-2 variants with broad neutralization. Proceedings of the National Academy of Sciences of the United States of America, 119, e2122954119.
13. Webb,B. and Sali,A. (2016) Comparative protein structure modeling using MODELLER. Current Protocols in Bioinformatics, 54, 5–6.
14. Pancotti,C., Benevenuta,S., Birolo,G., Alberini,V., Repetto,V., Sanavia,T., Capriotti,E. and Fariselli,P. (2022) Predicting protein stability changes upon single-point mutation: A thorough comparison of the available tools on a new dataset. Briefings in Bioinformatics, 23, bbab555.
15. Laimer,J., Hiebl-Flach,J., Lengauer,D. and Lackner,P. (2016) MAESTROweb: a web server for structure-based protein stability prediction. Bioinformatics, 32, 1414–1416.
16. Benevenuta,S., Pancotti,C., Fariselli,P., Birolo,G. and Sanavia,T. (2021) An antisymmetric neural network to predict free energy changes in protein variants. Journal of Physics D: Applied Physics., 54, 245403.
17. Montanucci,L., Capriotti,E., Frank,Y., Ben-Tal,N. and Fariselli, P. (2019) DDGun: an untrained method for the prediction of protein stability changes upon single and multiple point variations. BMC Bioinformatics, 20, 1–10.
18. Savojardo,C., Fariselli,P., Martelli,P.L. and Casadio,R. (2016) INPS-MD: a web server to predict stability of protein variants from sequence and structure. Bioinformatics, 32, 2542–2544.
19. Rodrigues,C.H., Pires,D.E. and Ascher,D.B. (2018) DynaMut: predicting the impact of mutations on protein conformation, flexibility and stability. Nucleic Acids Research, 46, W350–W355.
20. Dehouck,Y., Kwasigroch,J. M., Gilis,D. and Rooman, M. (2011) PoPMuSiC 2.1: a web server for the estimation of protein stability changes upon mutation and sequence optimality. BMC Bioinformatics, 12, 1–12.
21. Pires,D.E., Ascher,D.B. and Blundell,T.L. (2014) DUET: a server for predicting effects of mutations on protein stability using an integrated computational approach. Nucleic Acids Research, 42, W314–W319.
22. Worth,C.L., Preissner,R. and Blundell,T.L. (2011) SDM—a server for predicting effects of mutations on protein stability and malfunction. Nucleic Acids Research, 39, W215–W222.
23. Chen,Y., Lu,H., Zhang,N., Zhu,Z., Wang,S. and Li,M. (2020) PremPS: Predicting the impact of missense mutations on protein stability. PLoS Computational Biology, 16, e1008543.
24. Li,B., Yang,Y.T., Capra,J.A. and Gerstein,M.B. (2020) Predicting changes in protein thermodynamic stability upon point mutation with deep 3D convolutional neural networks. PLoS Computational Biology, 16, e1008291.
25. Pires,D.E., Ascher,D.B. and Blundell,T.L. (2014) mCSM: predicting the effects of mutations in proteins using graph-based signatures. Bioinformatics, 30, 335–342.
26. Capriotti,E., Fariselli,P. and Casadio, R. (2005) I-Mutant2. 0: predicting stability changes upon mutation from the protein sequence or structure. Nucleic Acids Research, 33, W306–W310.
27. Li,Y., Huang,Y., Swaminathan,C.P., Smith-Gill,S.J. and Mariuzza,R.A. (2005) Magnitude of the hydrophobic effect at central versus peripheral sites in protein-protein interfaces. Structure, 13, 297–307.
28. Hanson,W.M., Domek,G.J., Horvath,M.P. and Goldenberg,D.P. (2007) Rigidification of a flexible protease inhibitor variant upon binding to trypsin. Journal of Molecular Biology, 366, 230–243.
29. Helland,R., Otlewski,J., Sundheim,O., Dadlez,M. and Smalås,A.O. (1999) The crystal structures of the complexes between bovine β-trypsin and ten P1 variants of BPTI. Journal of Molecular Biology, 287, 923–942.
30. Castro,M.J.M. and Anderson,S. (1996) Alanine point-mutations in the reactive region of bovine pancreatic trypsin inhibitor: effects on the kinetics and thermodynamics of binding to β-trypsin and α-chymotrypsin. Biochemistry, 35, 11435–11446.
